# Geographical emergence of sulfadoxine-pyrimethamine drug resistance-associated *P. falciparum* and *P. malariae* alleles in co-existing *Anopheles* mosquito and asymptomatic human populations across Cameroon

**DOI:** 10.1128/aac.00588-23

**Published:** 2023-11-10

**Authors:** Francis N. Nkemngo, Lymen W. Raissa, Daniel N. Nguete, Cyrille Ndo, Jerome Fru-Cho, Flobert Njiokou, Samuel Wanji, Charles S. Wondji

**Affiliations:** 1 Centre for Research in Infectious Diseases (CRID), Yaoundé, Cameroon; 2 Department of Microbiology and Parasitology, Faculty of Science, University of Buea, Buea, Cameroon; 3 Department of Biological Sciences, Faculty of Medicine and Pharmaceutical Sciences, University of Douala, Douala, Cameroon; 4 Research Foundation in Tropical Diseases and Environment, Buea, Cameroon; 5 Centre for Infection Biology and Translational Research, Forzi Institute, Buea, Cameroon; 6 Vector Biology Department, Liverpool School of Tropical Medicine, Liverpool, United Kingdom; The Children's Hospital of Philadelphia, Philadelphia, Pennsylvania, USA

**Keywords:** *P. falciparum*, *P. malariae*, sulfadoxine-pyrimethamine, drug resistance, *Anopheles* mosquitoes, humans, Cameroon

## Abstract

Malaria molecular surveillance remains critical in detecting and tracking emerging parasite resistance to anti-malarial drugs. The current study employed molecular techniques to determine *Plasmodium* species prevalence and characterize the genetic diversity of *Plasmodium falciparum* and *Plasmodium malariae* molecular markers of sulfadoxine-pyrimethamine resistance in humans and wild *Anopheles* mosquito populations in Cameroon. *Anopheles* mosquito collections and parasitological survey were conducted in villages to determine *Plasmodium* species infection, and genomic phenotyping of anti-folate resistance was accomplished by sequencing the dihydrofolate-reductase (*dhfr*) and dihydropteroate-synthase (*dhps*) genes of naturally circulating *P. falciparum* and *P. malariae* isolates. The malaria prevalence in Elende was 73.5% with the 5–15 years age group harboring significant *P. falciparum* (27%) and *P. falciparum + P. malariae* (19%) infections. The polymorphism breadth of the pyrimethamine-associated *Pfdhfr* marker revealed a near fixation (94%) of the triple-mutant -A^16^**I^51^R^59^N^108^**I^164^. The *Pfdhps* backbone mediating sulfadoxine resistance reveals a high frequency of the **V^431^A^436^G^437^**K^540^A^581^A^613^ alleles (20.8%). Similarly, the *Pmdhfr* N^50^K^55^**L^57^R^58^**S^59^S^114^F^168^I^170^ haplotype (78.4%) was predominantly detected in the asexual blood stage. In contrast, the *Pmdhps*– **S**^436^**A**^437^occured at 37.2% frequency. The combined quadruple N^50^K^55^**L^57^R^58^**S^59^S^114^F^168^I^170^_ **S^436^G^437^**K^540^A^581^A^613^ (31.9%) was the major circulating haplotype with similar frequency in humans and mosquitoes. This study highlights the increasing frequency of the *P. malariae* parasite mostly common in asymptomatic individuals with apparent *P. falciparum* infection. Interventions directed at reducing malaria transmission such as the scaling-up of SP are favoring the emergence and spread of multiple drug-resistant alleles between the human and mosquito host systems.

## INTRODUCTION

Malaria in Africa is principally caused by *Plasmodium falciparum* with *Plasmodium malariae*, *Plasmodium ovale*, and *Plasmodium vivax* considered as minority species. Efforts to control and eliminate malaria have been accelerated by anti-malarial drugs with control interventions such as intermittent preventive treatment in pregnancy (IPTp), perennial malaria chemoprevention (PMC), and seasonal malaria chemoprevention (SMC) employing anti-folates notably sulfadoxine-pyrimethamine (SP) as a chemoprophylaxis regimen for *P. falciparum* infections in many areas of Africa (1). Chemoprevention is known to be a generally safe and cost-effective strategy for targeting the most vulnerable populations to malaria. This strategy has yielded striking success in shrinking the global malaria mortality curve, particularly for pregnant women and infants (2, 3). However, the overreliance and indiscriminate use of the drug have been major multiplying factors for drug resistance which present an obstacle in malaria control where it has contributed to the rebound in malaria cases in Africa from 211,000,000 cases and 577,000 deaths in 2015 to 234,000,000 cases and 609,000 deaths in 2022 (1). Recent reports by the World Health Organization (WHO) indicate that the emergence and spread of *Plasmodium* parasite resistance by *Anopheles* mosquitoes threatens to reverse the gains achieved in malaria control over the past decade (1, 4). The increasing occurrence and spread of drug resistant alleles render a threat to malaria treatment efficacy in pregnancy and SMC in which SP remains the frontline intervention in Africa, thus emphasizing the need for continuous monitoring and surveillance (5).

The anti-folate SP is a slow-acting combination drug with a prolonged elimination half-life of up to 14 days in blood, usually employed as a chemoprophylaxis strategy against malaria. The drug inhibits two key enzymes in the *P. falciparum* folate metabolic pathway notably dihydrofolate reductase (*dhfr*) and dihydropteroate synthase (*dhps*) (6). Failure of SP therapy is associated with the accumulation of point mutations in these two parasite genes, and mutations in the genes encoding these enzymes have been validated as causal variants of SP drug resistance in field parasite isolates (7). The landscape of *P. falciparum* resistance to pyrimethamine is mediated by a stepwise progressive selection of a series of non-synonymous point mutations on codons 50, 51, 59, and 164 originating from the ancestral S108N mutant allele. Parasites harboring these N^51^I-C^59^R-S^108^N triple-mutant alleles often exhibit marked reduction to pyrimethamine susceptibility *in vitro* with a higher risk of SP treatment failure in clinical surveys (8). Moreover, the presence of an additional I164L quadruple mutant is known to confer complete SP clinical failure as observed in various studies across Africa, Southeast Asia, and Latin America (9). Similarly, *P. falciparum* resistance to sulfadoxine is primarily driven by the presence of the A437G and K540E codon mutations in the *dhps* backbone. This becomes programmatically relevant for malaria control as the increasing frequency of the quintuple mutation (*Pfdhfr* IRN + *Pfdhps* GE) in local parasite populations is predictive of complete SP clinical tolerance (10). Key mutations in the *dhfr* and *dhps* genes have been exploited as molecular markers for mapping, inferring, and tracking the frequency of circulating drug-resistant parasite haplotypes in real time and geography (11). Based on this, the WHO recommends that the use of IPTp and PMC with SP be implemented based on a >50% frequency threshold of the *dhps* 437G + 540E allele (5, 12), further emphasizing the need for routine geographical surveillance to guide malaria intervention policy decisions.

While an enormous data set exists for *P. falciparum*, there is a paucity of information on the drug resistance polymorphism profile of *P. malariae* to conventional SP drugs. This becomes very important as increasing epidemiological evidence is bringing to prominence the occurrence of *P. malariae* as an important parasite species often co-niching with *P. falciparum* in high-malaria transmission settings such as Cameroon (11). Unfortunately, research on *P. malariae* biology remains largely neglected and lagging because it is less frequently recognized to account for malaria cases although PCR detection has identified this parasite species to be more common and persistent in recent years than previously thought (13). Consequently, it is empirical to determine whether SP pressure has selected *dhfr* and *dhps* genetic variants in circulating *P. malariae* populations since chronic, low-density asymptomatic infection is a successful biological adaptation unique to this species (14). Although *P. malariae* infections are not usually treated directly with SP, the high frequency of mixed infections with *P. falciparum* and non-falciparum species common in malaria-endemic settings implies that a large genome pool of *P. malariae* parasites has inevitably been exposed to indiscriminate SP therapy. This ultimately creates an enabling environment for the emergence of drug-resistant alleles that may compromise treatment success.

In the quest of mapping resistance epidemiology, *Plasmodium* drug resistance typing has traditionally relied on the blood stage of the parasite in the human host without much attention to the mosquito vector (15, 16). Limited data exist on *Plasmodium* midgut and salivary gland life cycle stages in the mosquito which otherwise constitutes a key developmental phase responsible for sexual fertilization and parasite genetic recombination. Moreover, allele frequency may differ between the two host systems due to super-infection, multiplicity of infection, and immune clearance in humans, as well as bottleneck checkpoints, genetic recombination, and fitness cost in mosquitoes which could impact the disparate transmissibility of mutant parasite variants. Thus, this study characterizes the genetic determinants of the *dhfr* and *dhps* backbones involved in SP resistance in *P. falciparum* and *P. malariae* parasites in the *Anopheles* and human host systems.

## RESULTS

### Molecular identity of *Anopheles* vectors and *Plasmodium* species infection rates

A total of 6,529 adult female mosquitoes belonging to the *Anopheles* genus were collected indoor across nine localities in Cameroon as previously documented (11). Molecular composition of the Anopheline fauna in these sites revealed three major species including *Anopheles funestus s.s* (Elende, Elon, Mibellon, and Obout), *An. gambiae s.s* (Bankeng, Mangoum), and *An*. *coluzzii* (Bonaberi, Gounougou, and Simatou). The analysis of the head and thorax (H/T) and midgut abdomen (Abd) portion of the *Anopheles* mosquitoes across the 09 localities reveals a varied stage developmental *Plasmodium* infection rate as previously published (11). The sporozoite infection rate (SIR) was between 0.4% (*An. coluzzii*) and 13.8% (*An. funestus*). *P. falciparum* was predominant (67.7%–100%) followed by *P. malariae* (4.6%–26.5%) at the H/T level. Similarly, a higher occurrence of *Plasmodium*-infected midgut isolates was recorded at a frequency between 1.1% (*An. coluzzii*) and 25% (*An. funestus*). Species-specific prevalence ranged between 60.2% and 100%, 0% and 37.6%, and 0% and 5.6% for *P. falciparum*, *P. malariae*, and *P. ovale*, respectively (11). *An. funestus* exhibited the highest infection rates in both separated mosquito body sections.

### Characteristics of study participants, malaria prevalence, and *Plasmodium* spp. parasite density in elende

**Fig 1:**
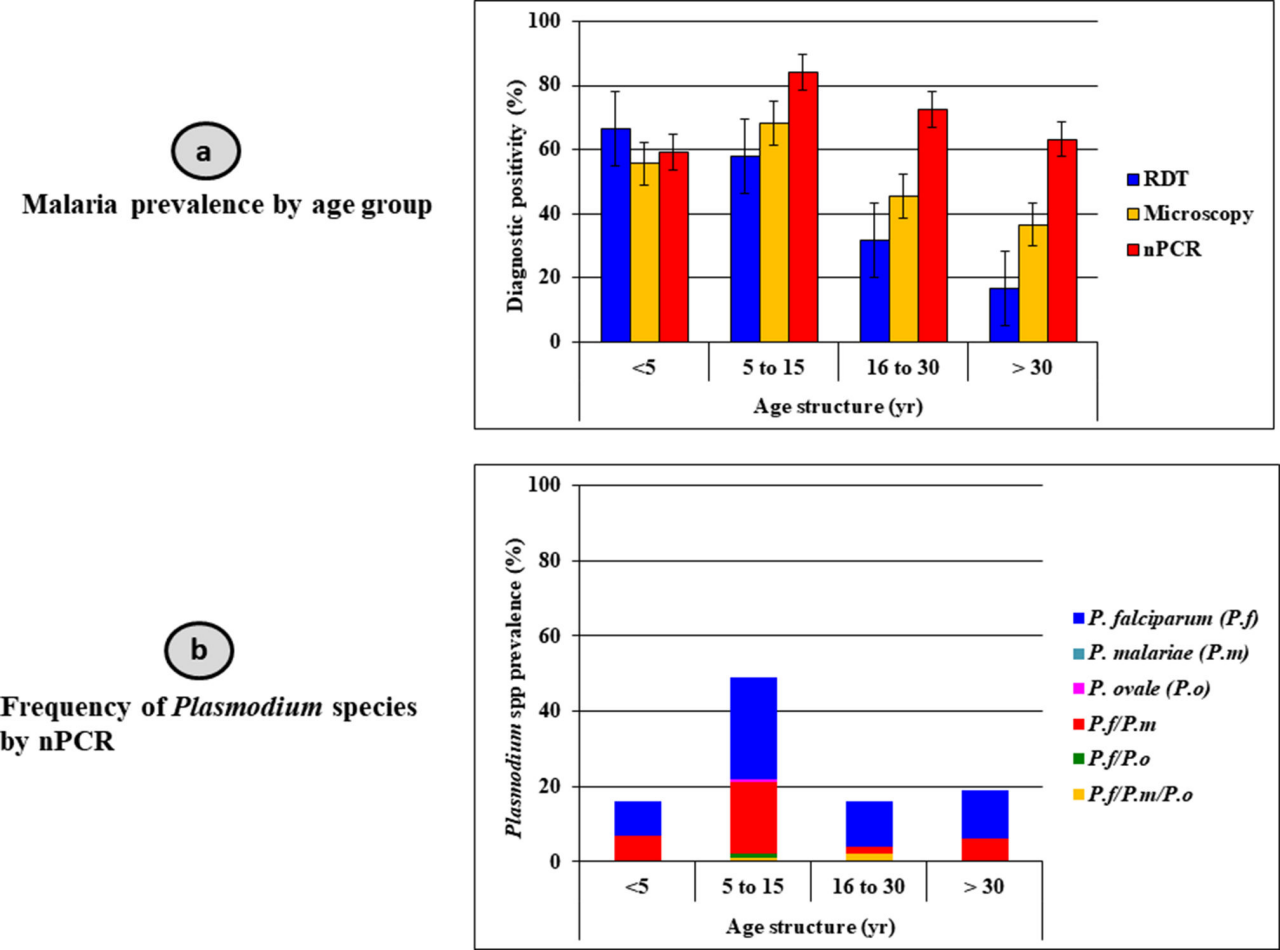
(a) Age stratification of malaria prevalence and (b) *Plasmodium* speciation in Elende.

The baseline characteristics of the study population in Elende are shown in Table 1. A total of 136 subjects aged between 1 year and 73 years participated in the study. Females (66.9%) had a higher enrollment count than males (33.1%), with the 5–15 years (41.9%) being the dominant age group. Malaria prevalence rates of 55.1% (75), 46.3% (63), and 73.5% (100) were recorded by microscopy, rapid diagnostic test (RDT), and nested polymerase chain reaction (nPCR), respectively (Fig. 1). Comprised of an asymptomatic infection pattern with a mean temperature of 36.6°C, microscopic examination reveals a generally high frequency of *P. falciparum* (*Pf* = 74.7%) followed by mixed *P. falciparum + P. malariae* (*Pf*/*Pm*) species (21.3%), disproportionately affecting the 5–15 years group (40% *Pf* and 10.7% *Pf*/*Pm*) (χ^2^ = 19.7, *P* = 0.001). Similarly, PCR reported an overall high positivity of *P. falciparum* (61%) and mixed *Pf + Pm* (34%) with the burden concentrated on the 5–15 years cohort as in Fig. 1 and Table 1. The overall trophozoite density [(*Pf* = 1,966 trophs/µL; *Pf + Pm* (3,601 *Pf* trophs/µL and 685.4 *Pm* trophs/µL)] and gametocyte carriage [(*Pf*: 48 gametocytes/µL; *Pf + Pm* (16 *Pf* gametocytes/µL and 53.1 *Pm* gametocytes/µL)] were significantly higher (*P* = 0.01) among children < 5 years than their counterpart.

**TABLE 1:** Malaria prevalence and multispecies *Plasmodium* infection frequency in Elende

Parameter	Age groups (yr); % (*n*)				Gender		Total
	< 5	5–15	16–30	>30	Female	Male	
% (N)	19.9 (27)	41.9 (57)	16.2 (22)	22.1 (30)	66.9 (91)	33.1 (45)	136
Mean age (yr)	2.4	9.2	21.2	46.6	19.04	16.3	18.0
Mean height (m)	0.85	1.3	1.6	1.6	1.3	1.3	1.3
Mean weight (kg)	12	28	57.5	70	42.2	38.3	40.9
Mean temperature (°C)	36.3	36.4	36.5	36.3	36.6	36.7	36.6
Fever	3.7 (1)	0 (0)	0 (0)	0 (0)	1.1 (1)	0 (0)	0.7 (1)
Malaria prevalence (%, *n*)							
RDT	66.7 (18)	57.9 (33)	31.8 (7)	16.7 (5)	37.4 (34)	64.4 (29)	46.3 (63)
Microscopy	55.6 (15)	68.4 (39)	45.5 (10)	36.7 (11)	50.6 (46)	64.4 (29)	55.1 (75)
PCR	59.3 (16)	85.9 (49)	72.7 (16)	63.3 (19)	68.1 (62)	84.4 (38)	73.5 (100)
*Plasmodium* species prevalence (%, *n*)							
Microscopy							
*P. falciparum (Pf*)	13.3 (10)	40 (30)	12 (9)	9.3 (7)	76.1 (35)	72.4 (21)	41.2 (56)
*P. malariae (Pm*)	0 (0)	1.3 (1)	1.3 (1)	0 (0)	4.3 (2)	0 (0)	1.5 (2)
*P. ovale (Po*)	0 (0)	0 (0)	0 (0)	0 (0)	0 (0)	0 (0)	0 (0)
*Pf/Pm*	5.3 (4)	10.7 (8)	1.3 (1)	4 (3)	17.4 (8)	27.6 (8)	21.3 (16)
*Pf/Pm/Po*	0 (0)	1.3 (1)	0 (0)	0 (0)	2.2 (1)	0 (0)	1.3 (1)
PCR							
*Pf*	9 (9)	27 (27)	12 (12)	13 (13)	66.1 (41)	52.6 (20)	61 (61)
*Pm*	0 (0)	0 (0)	0 (0)	0 (0)	0 (0)	0 (0)	0 (0)
*Po*	0 (0)	1 (1)	0 (0)	0 (0)	1.6 (1)	0 (0)	1 (1)
*Pf/Pm*	7 (7)	19 (19)	2 (2)	6 (6)	30.7 (19)	39.5 (15)	34 (34)
*Pf/Po*	0 (0)	1 (1)	0 (0)	0 (0)	1.6 (1)	0 (0)	1 (1)
*Pf/Pm/Po*	0 (0)	1 (1)	2 (2)	0 (0)	0 (0)	7.9 (3)	3 (3)
Geometric mean trophozoite (parasites/µL)							
*Pf*	2788	2017	2542	706.2	1826	2221	1966
*Pm*	0 (0)	160	760	0 (0)	348.7	0 (0)	348.7
*Pf + Pm*	6384	4847	0 (0)	1393	3827	3438	3601
*P.f P.m*	928.6	655	0 (0)	590.9	626.7	616.8	685.4
Mean gametocyte (parasites/µL)							
*Pf*	48	48	0 (0)	0 (0)	33.3	144	48
*Pm*	64	27.7	0 (0)	0 (0)	36.6	32	34.7
*Pf + Pm*	0 (0)	16	0 (0)	0 (0)	16	16	16
*P.f P.m*	0 (0)	53.1	0 (0)	0 (0)	176	16	53.1

### Validity of CareStart for malaria diagnosis and concurrence with microscopy and PCR

The CareStart Malaria HRP2 pf Ag RDT had a sensitivity of 59.6% [95% confidence interval (CI): 51.2%–68.0%] and specificity of 89.2% (95% CI: 83.6%–94.8%). The positive predictive value (PPV), negative predictive value (NPV), and accuracy (acc) were 93.7% (95% CI: 88.4%–99%), 45.2% (95% CI: 34.1%–56.3%), and 83.6% (95% CI: 77.6%–89.6%), respectively. Using PCR as a reference, the false-positive RDT result was 2.9% (04) while the false-negative rate was 29.4% (40). The measure of agreement, kappa (k) between microscopy, and CareStart Malaria HRP2 pf Ag RDT was 0.6.

### Comparison of *Dhfr* and *Dhps* Genes between *P*. *malariae* and *P. falciparum*

Nucleotide alignment of indigenous Cameroonian *Pmdhfr* and *Pmdhps* isolates with the equivalent *P. falciparum dhfr* and *dhps* GenBank reference sequences reveals a sequence homology score of 72% and 80%, respectively. Similarly, the A/T nucleotide base content for both parasite species is 75% for *Pfdhfr*, 75% for *Pfdhps*, 73% for *Pmdhfr*, and 75% for *Pmdhps,* suggesting a high-mutation plasticity rate.

### Polymorphism profile and genetic diversity statistics of *Pfdhfr* and *Pfdhps* gene sequences from *P. falciparum*-infected *Anopheles* mosquitoes and human populations

**Fig 2:**
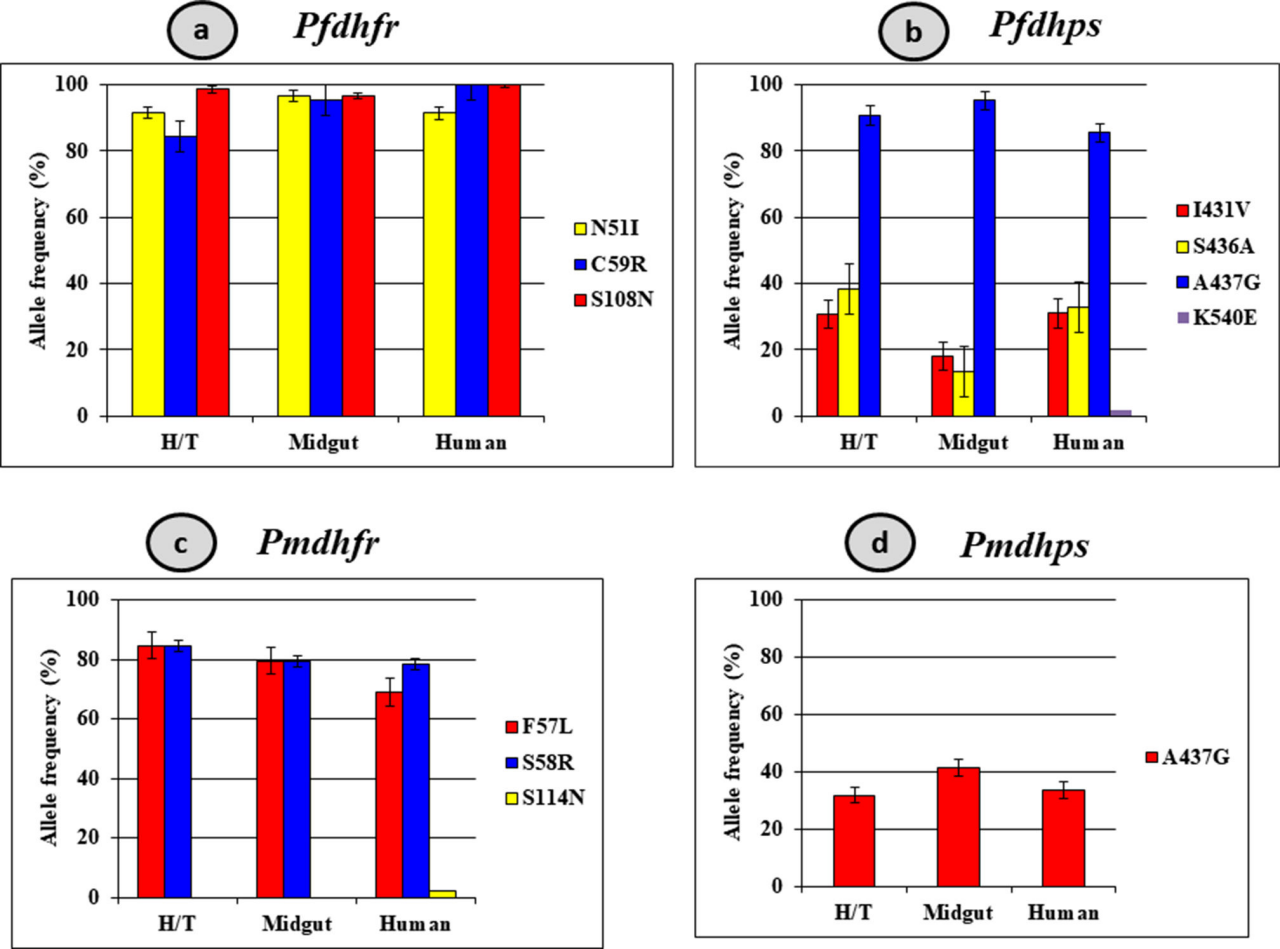
Frequency of sulfadoxine-pyrimethamine mutant alleles in circulating *P. falciparum* and *P. malariae* parasite isolates in the mosquito and human host systems. (**A**) *Pfdhfr*, (**B**), *Pfdhps*, (**C**), *Pmdhfr*, and (**D**)*Pmdhps.*

**Fig 3:**
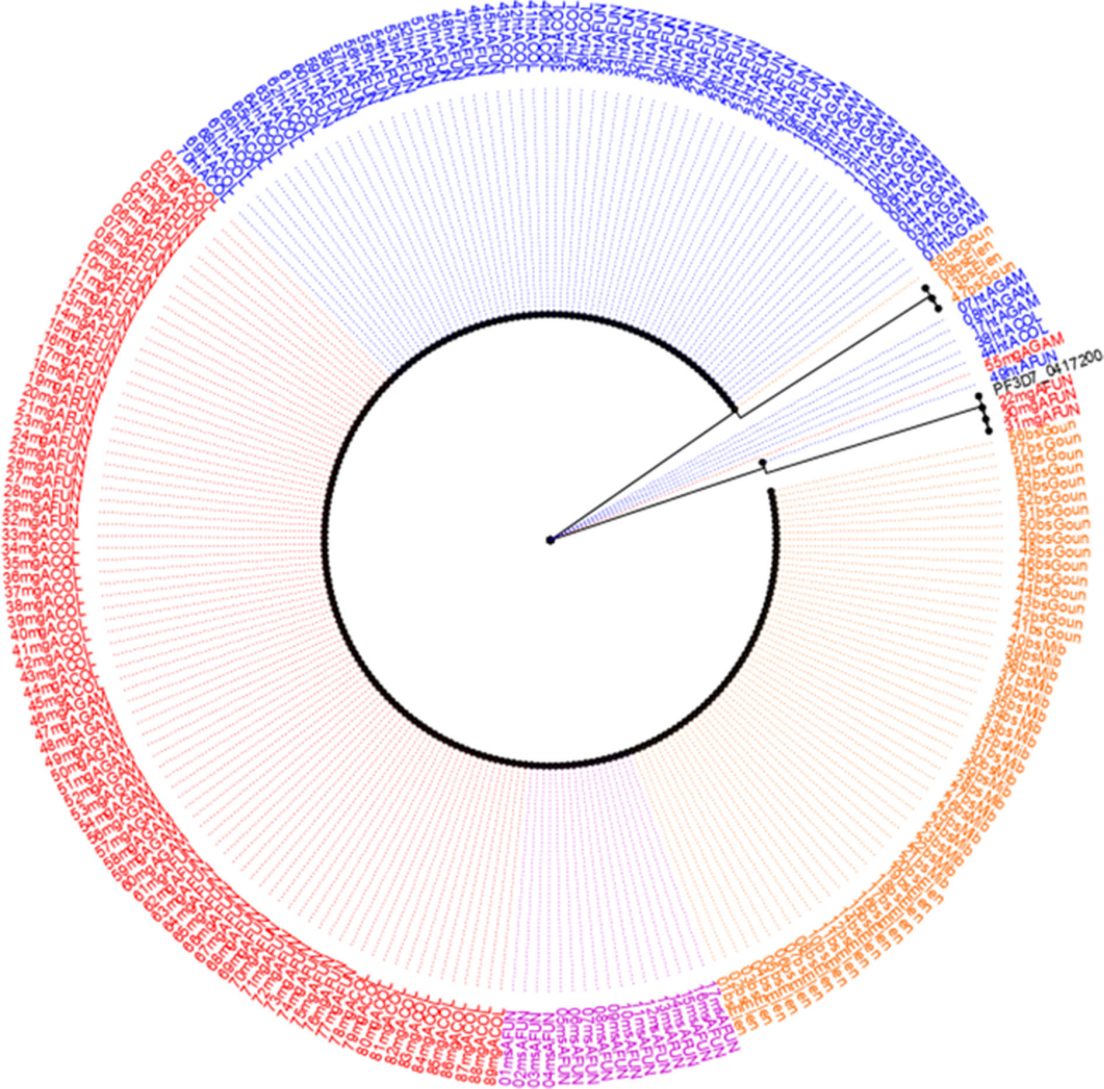
Phylogenetic map of the sequenced *Pfdhfr* isolates revealing homogenous parasite cluster.

**Fig 4:**
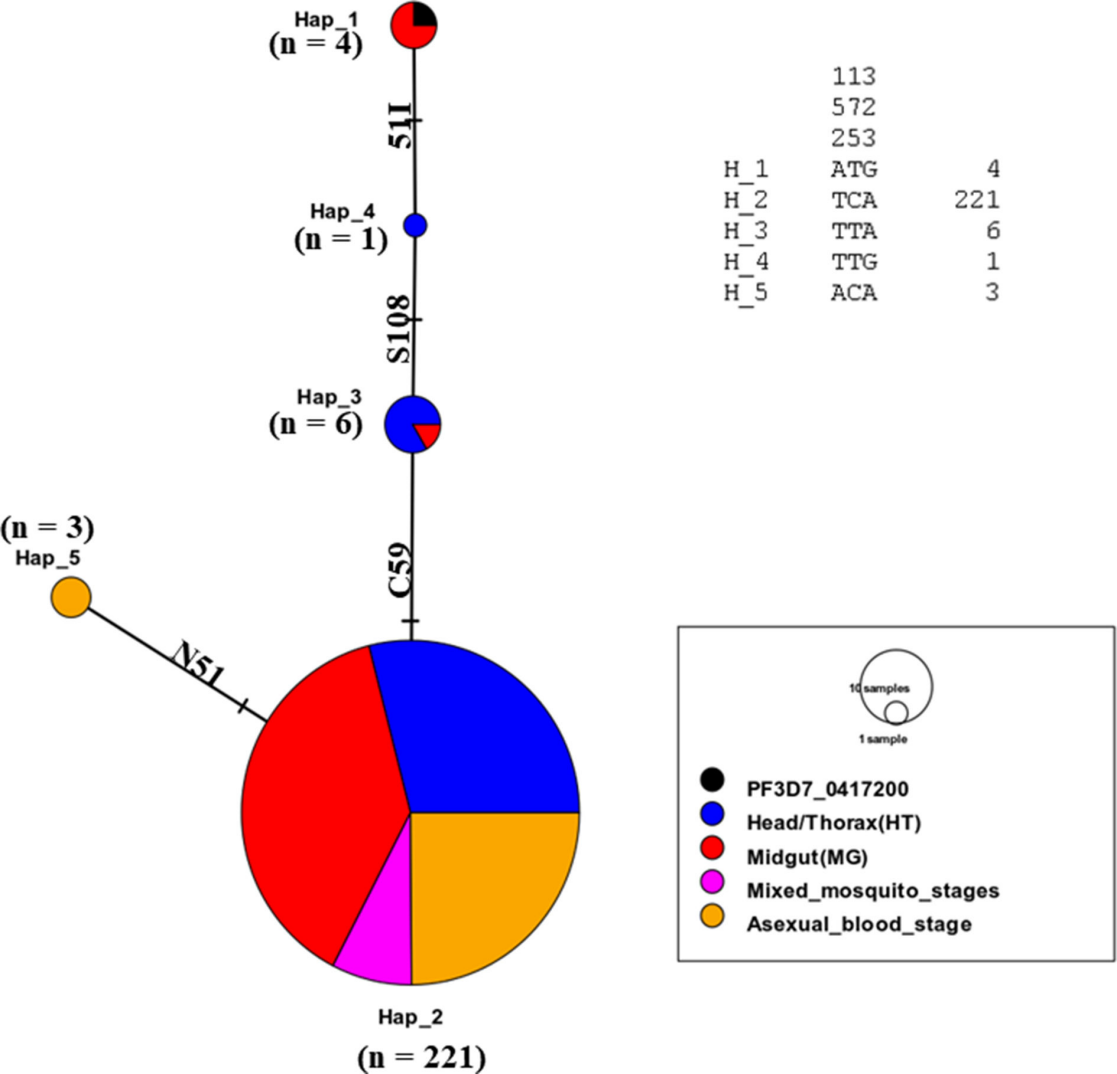
Haplotype network of the sequenced *Pfdhfr* isolates identifying a dominant mutant population.

A 635-bp fragment of the *dhfr* gene (spanning nucleotide 40 to 633) was amplified from the *P. falciparum* parasite stages in the *Anopheles* mosquito vector and the human host. A total of 234 samples (70 H/T isolates + 89 midgut isolates + 17 mixed mosquito stages + 58 human blood stage) were sequenced (Table S1). Combined multiple sequence alignments with the PlasmoDB reference (PF3D7_0417200) reveal varying near-fixation frequency of singleton mutant alleles in drug resistance-associated loci [N51**I** (93.2%); C59**R** (93.6%); S108**N** (98.3%)] in both the human and mosquito host systems (Fig. 2). Similarly, the frequencies of the double-mutant alleles -A^16^**I^51^**C^59^**N^108^**I^164^ and - A^16^N^51^**R^59^N^108^**I^164^- were 2.9% (*n* = 7) and 1.3% (*n* = 3), respectively; and a triple A**IRN**I allele frequency of 94.4% (*n* = 221) as shown in Table S1 to S5. The frequency of the triple-mutant alleles was similar between the human (94.8%) and mosquito stages (H/T: 91.4%, midgut: 95.5%). The wild-type allele (**ANCSI**) circulated at a generally low prevalence of 3.4% predominantly in the midgut (*n* = 3/89) as outlined in Table S5. No mutant alleles were detected at codons 16 and 164 in the *Pfdhfr* gene. Genetic diversity statistics of aggregated *Pfdhfr* stage-specific population reveals five haplotypes, dominated by the mutant parasite cluster (H2) with a reduction in diversity (Hd = 0.115) and a negative Tajima value (*D* = −0.96879) (*P* > 0.10) as shown in Table S6. Maximum-likelihood phylogeny employing the Hasegawa-Kishino-Yano (HKY) model shows five clades (Fig. 3) principally sized by the mutant sequence types (H2: *n* = 221) followed by the wild (H1: *n* = 4) and minority isolates (H3 to H5) as seen in Fig. 4. There was no marginal difference (*P* = 0.83) in the haplotype diversity between distinct mosquito stages and human asexual forms.

**Fig 5:**
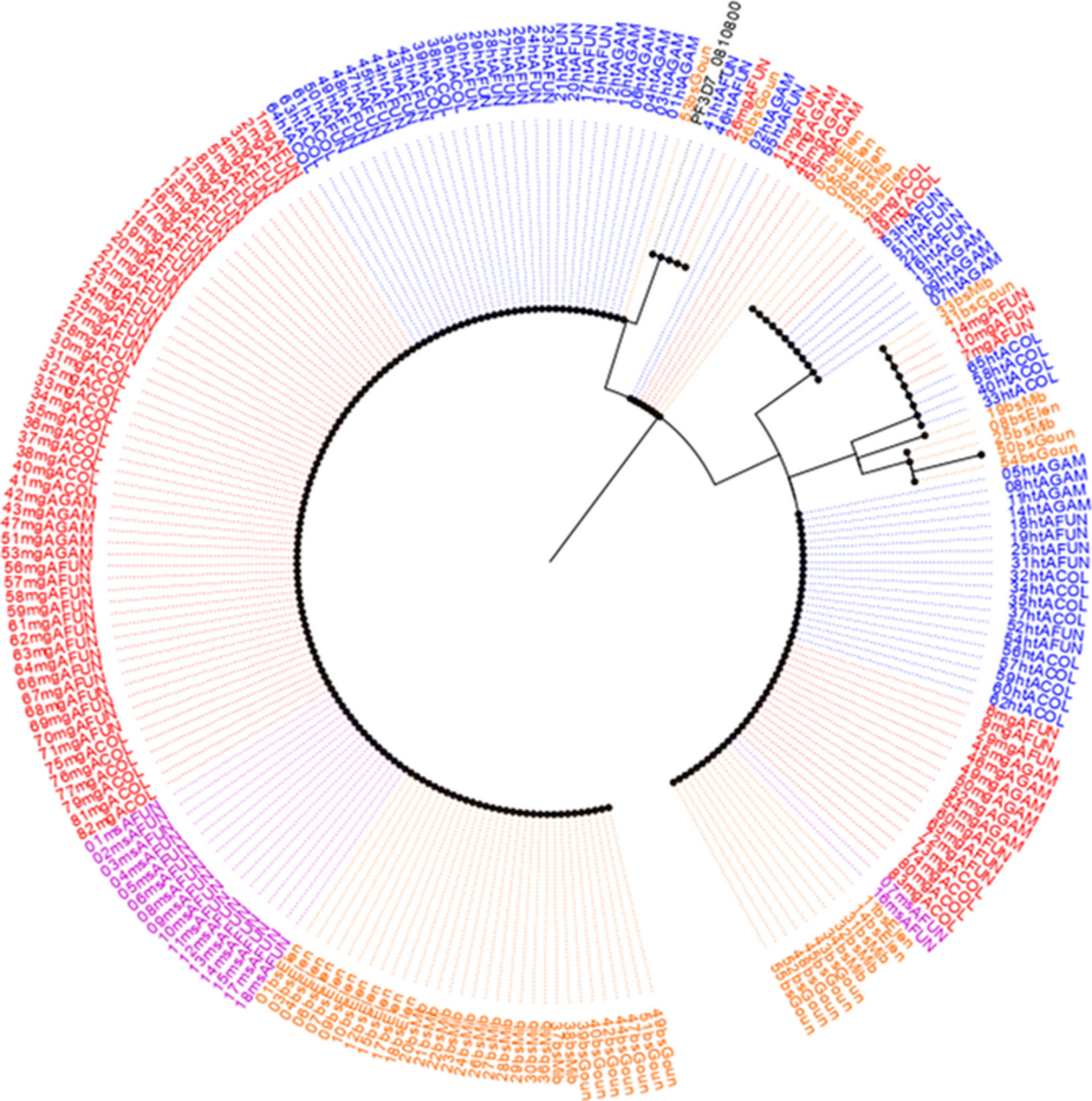
Phylogenetic plot of the sequenced *Pfdhps* isolates showing distinct population clusters.

**Fig 6:**
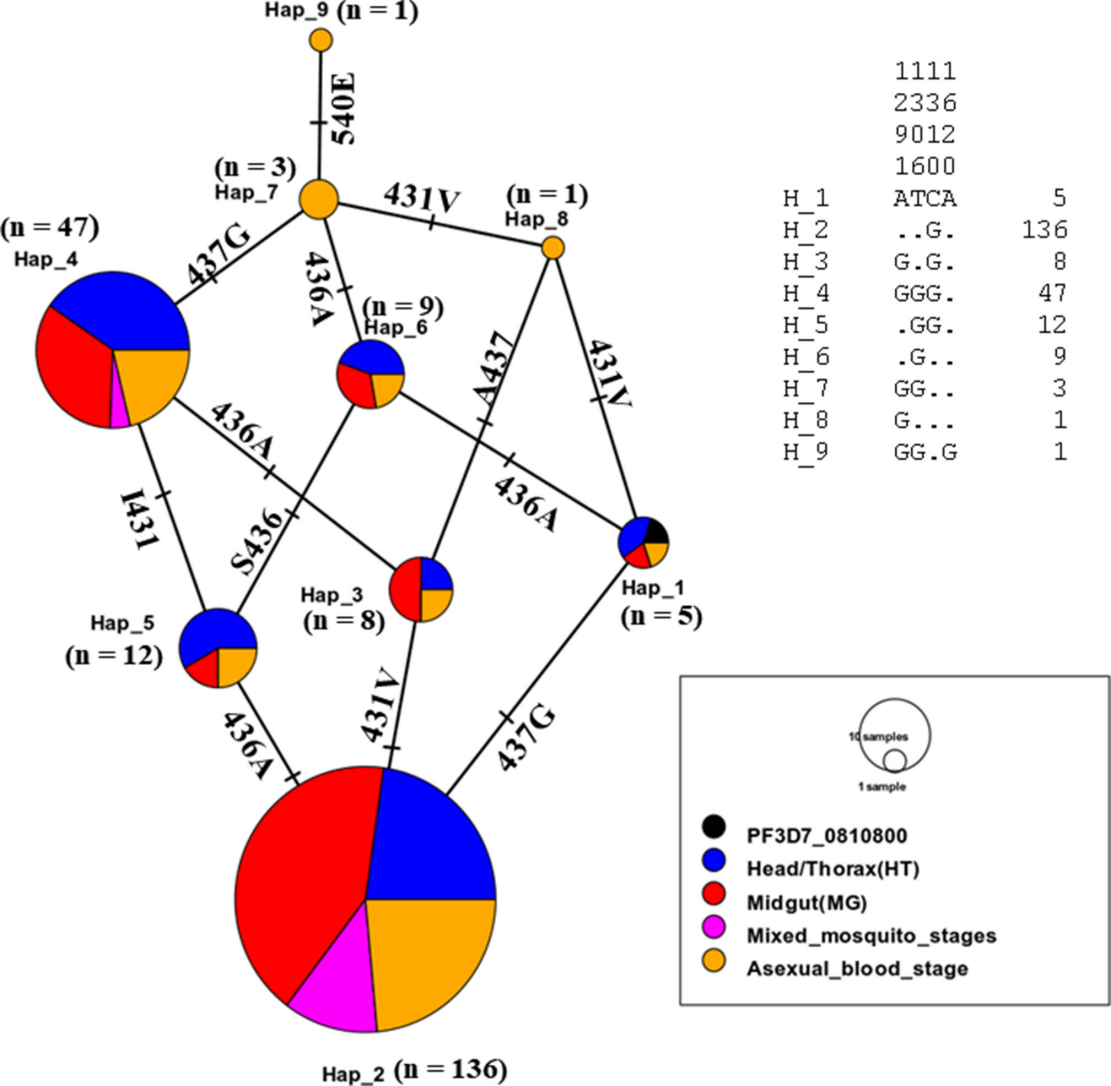
Haplotype network of the sequenced *Pfdhps* isolates identifying unique parasite assemblages.

Similarly, an amplified 720-bp *Pfdhps* gene fragment [covering nucleotide positions: 1267(codon 423) to 2044 (codon 681)] was sequenced across *P. falciparum* developmental stages in both mosquito and human host systems and matched with the reference isolate (Fig. 2–4). Multiple sequence alignment of 221 pooled *Pfdhps* sequences comprising 65 H/T isolates, 83 midgut isolates, 18 mixed mosquito stages, and 55 asexual blood stage isolates reveals an overall SNP frequency of 38.5% (*n* = 85) with three polymorphic sites (Table S2). This is characterized by the presence of a novel emerging I^431^**V** mutant allele (Fig. 2–4) in the H/T (32.3%, *n* = 21/65), midgut (24.1%, *n* = 20/83), mixed mosquito stages (11.1%, *n* = 2/18), and human blood stage (30.9, *n* = 17/55) isolates. The historically known S^436^**A** mutation was observed at a frequency of 46.2% (H/T), 25.3% (midgut), 11.1% (mixed mosquito stages), and 34.5% (human blood stage) while the A^437^**G** occurred at 90.8%, 95.2%, and 85.5% in the H/T, midgut, and human blood stages, respectively (Fig. 2; Table S2). Notably, the presence of mixed polymorphism for the *Pfdhps* alleles in the midgut stage suggests the probable outcrossing and genetic recombination event resulting from wild and mutant parasite allelic lineages. The triple mutant **V^431^A^436^G^437^** K^540^A^581^A^613^ was the most dominant allele in the H/T (29.2%), midgut (19.3%), and asexual blood stages (trophozoite) (16.4%) sequenced isolates. Similarly, the quadruple mutant -**V^431^A^436^G^437^****E^540^**A^581^A^613^ (0.5%) was found only in one sample blood stage parasite circulating in Gounougou. The wild-type I^431^S^436^A^437^K^540^A^581^A^613^ allele documented a prevalence of 3.1%, 1.2%, 0%, and 1.8% in the H/T, midgut, mixed mosquito, and asexual blood stages, respectively, with complete absence of mutations at codons 581 and 613 in the *Pfdhps* gene (Tables S2 to S5). Furthermore, Tamura three-parameter-based dendrogram plot and genetic diversity data confirm the circulation of 09 haplotypes (Fig. 5) with a diversity score of 0.58 and a positive Tajima *D* value of 0.874 (*P* > 0.10) (Table S7). A disparate parasite cluster was observed with the mutant sib-ship populations H2 (*n* = 136) and H4 (*n* = 47) exhibiting dominance distinct from the wild haplotype (H1). Minority mutants occupied the H2–H9 labels (Fig. 6). The haplotype diversity was similar for mosquito H/T (0.68) and the human blood stage (0.63) but lower for the midgut stage (0.49).

### Polymorphism frequency and genetic variability in the *Pmdhfr* and *Pmdhps* genes from *P. malariae*-infected *Anopheles* mosquitoes and humans

**Fig 7:**
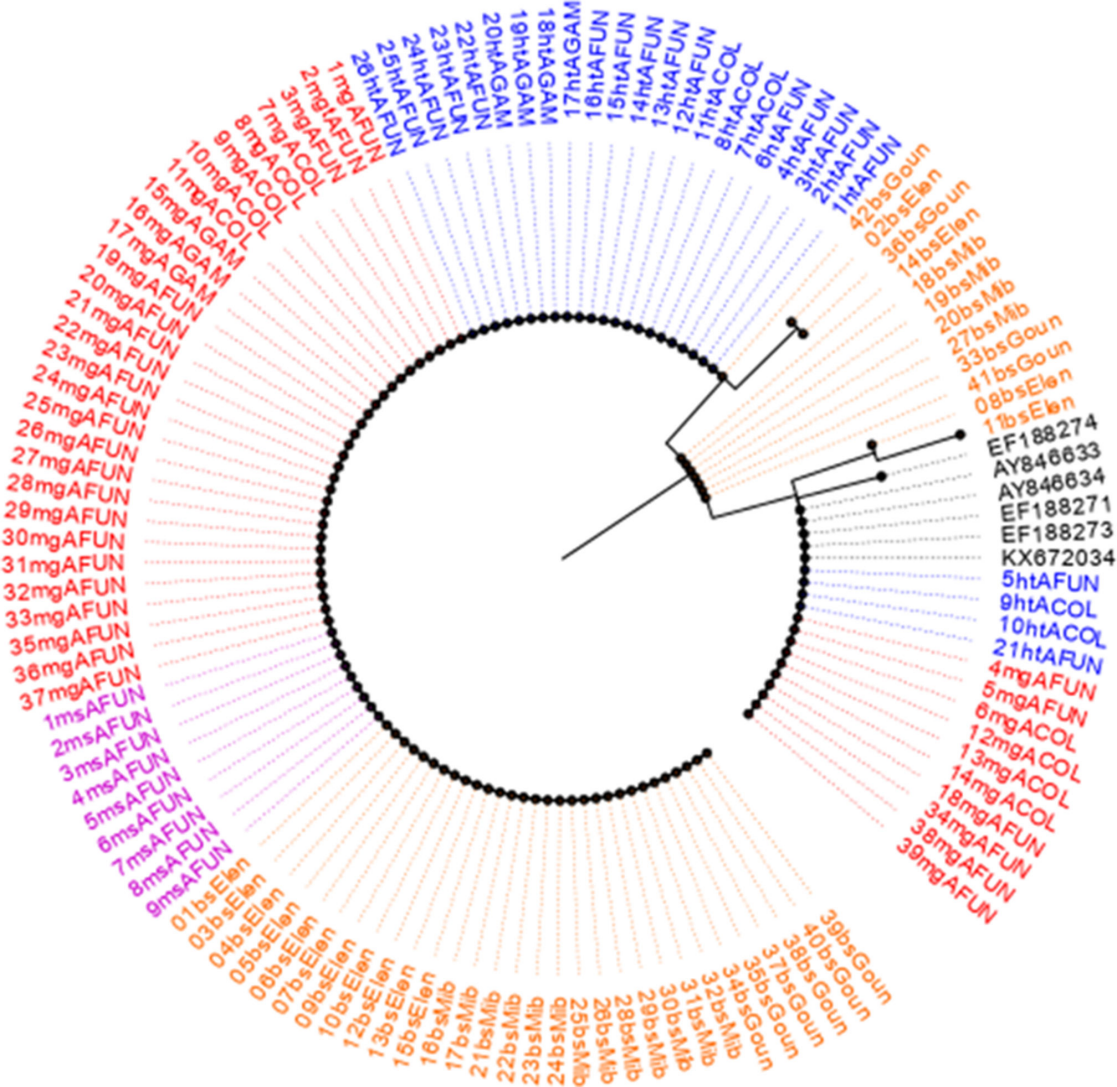
Phylogeny map of the sequenced *Pmdhfr* isolates revealing a high evolutionary relatedness of a major mutant clade.

**Fig 8:**
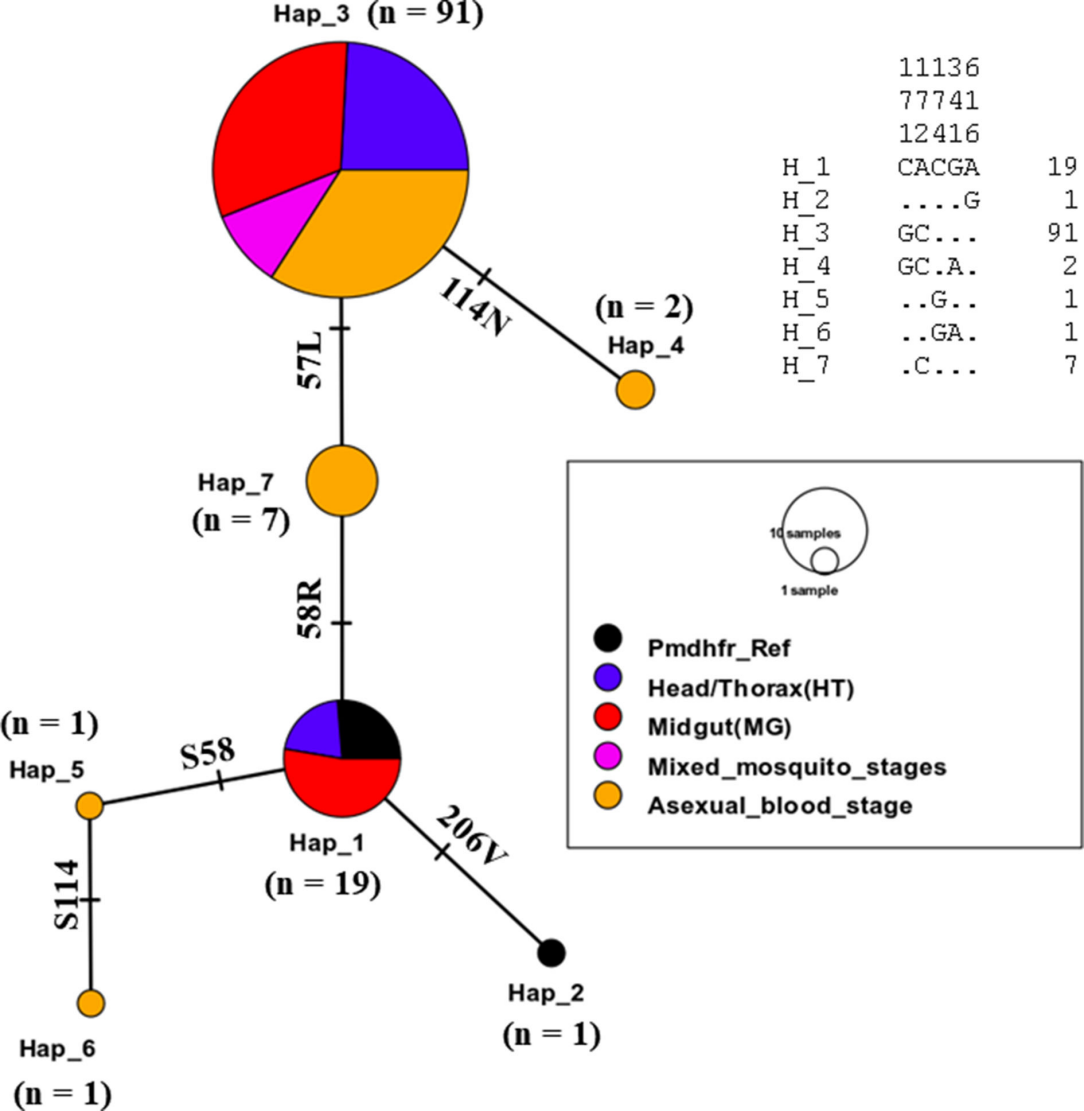
Haplotype network of the sequenced *Pmdhfr* isolates identifying two dominant mutant parasite populations.

A 650-bp fragment [spanning nucleotide bases: 133 (codon 45) to 674 (codon 224)] of the *Pmdhfr* gene was sequenced from 116 *P*. *malariae* isolates: 26 H/T, 39 midgut, 09 mixed mosquito, and 42 asexual blood stages. A total of 542 nucleotide sites comprising 538 monomorphic (invariable) and 04 polymorphic sites were found. Polymorphic sites were defined by the presence of 04 non-synonymous substitutions including F57**L** (^TTC^171**^TTG^**), S^58^**R** (^AGC^172**^CGC^**), S^58^**R** (^AGC^174**^CGG^**), and S^114^**N** (^AGC^341**^AAC^**) as in Fig. 3 and 4. The *P. malariae dhfr* amino acid residue substitutions, S^58^R and S^114^N, correspond to orthologous codons C^59^R and S^108^N, respectively, in the *Pfdhfr* gene. The overall frequency rates (Fig. 2 & Table S3) of the F^57^L, S^58^R, and S^114^N alleles were 80.2%, 81.9%, and 2.6%, respectively, categorized according to developmental stages H/T [F^57^L = 84.6% (22), S^58^R = 84.6% (22), S^114^*N* = 0%], midgut [F^57^L = 74.4% (29), S^58^R = 74.4% (29), S114*N* = 0%], mixed mosquito [F^57^L = 100% (9), S^58^R = 100% (9), S^114^*N* = 0%], and asexual blood stages [F^57^L = 78.6% (33), S^58^R = 100% (42), S^114^*N* = 7.1% (3)]. The single-mutant allele was observed only for the S^58^R (6.9%, 8/116) notably during the asexual blood stage-sequenced isolates. Heterozygous F^57^L (9.5%), S^58^R (16.7%), and S^114^N (4.8%) were predominantly present only in the asexual blood stage. A high proportion of paired double mutant, **L^57^R^58^**, was characteristic of the population at 77.6% (90/116). Isolates having the double **R^58^N^114^** and triple **L^57^R^58^N^114^** allele were found at minority frequency rates of 0.9% (1/116) and 1.7% (2/116), respectively, with 12.1% (14/116) wild-type sequence isolates observed (Table S5). Genetic diversity parameters show no difference in haplotype diversity among sequences between mosquito stages [H/T (Hd = 0.271 and midgut (Hd = 0.391)] and human stages (Hd = 0.434). Overall, six haplotypes with a negative Tajima score of −0.27137 (*P* > 0.10) were deduced from the sequence population (Table S8). The evolutionary history deduced from the maximum-likelihood method and Tamura 3-parameter model (Fig. 7) in concurrence with the haplotype network (Fig. 8) reveals seven distinct groups with the wild-type clustering with the majority of the Thailand isolates (H1; *n* = 19). The dominant H3 (*n* = 91) cluster consists of the autochthonous paired double-mutant assemblages at nucleotides 171 (F^57^L) and 172 (S^58^R). The low frequency of the S^114^N substitution (only present in the human blood stage) constituting the H4 + H6 haplotypes was observed to be slightly distant from the others.

**Fig 9:**
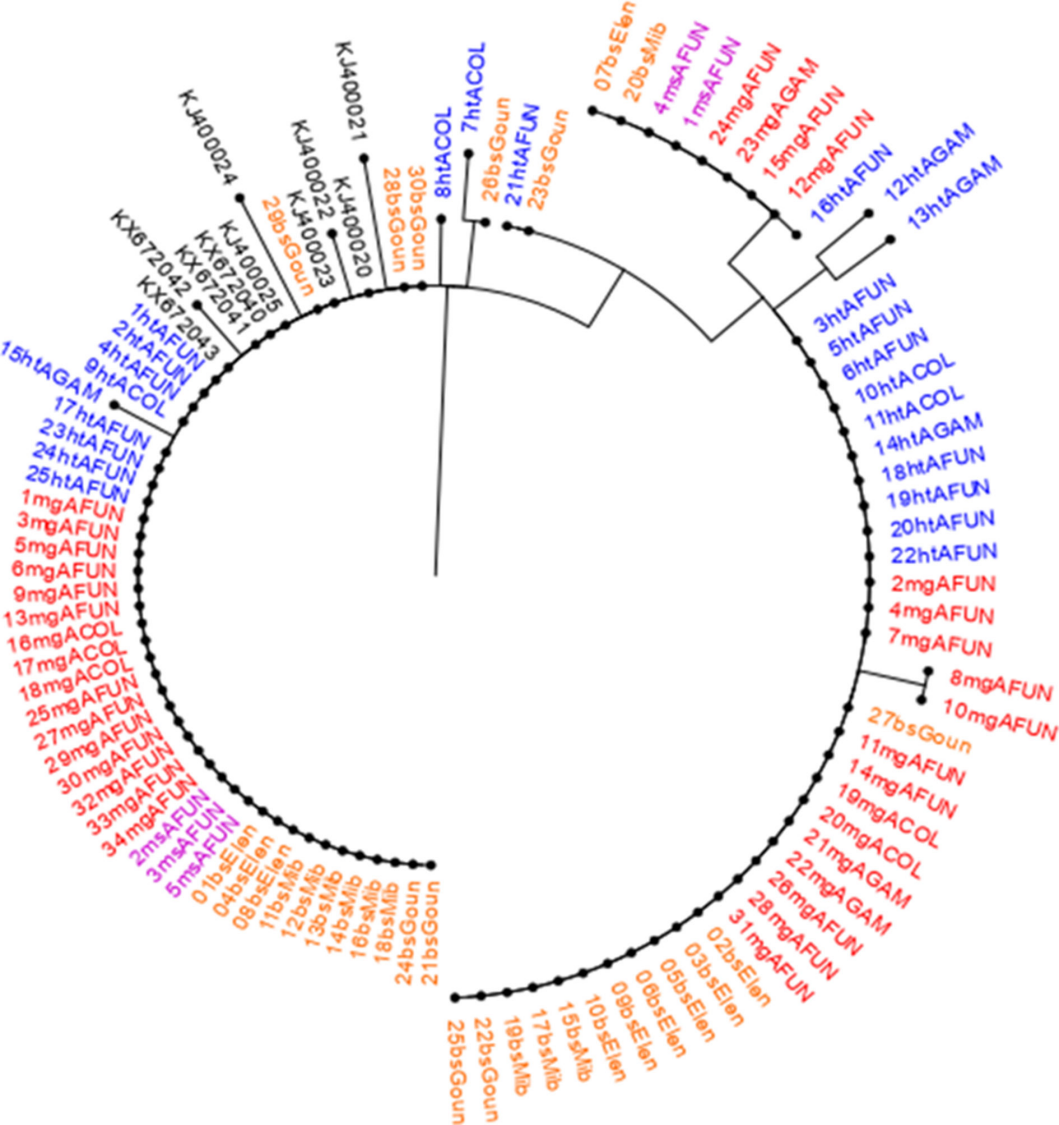
Dendrogram of the sequenced *Pmdhps* isolates revealing cluster relatedness of the mutant parasite population backbone.

**Fig 10:**
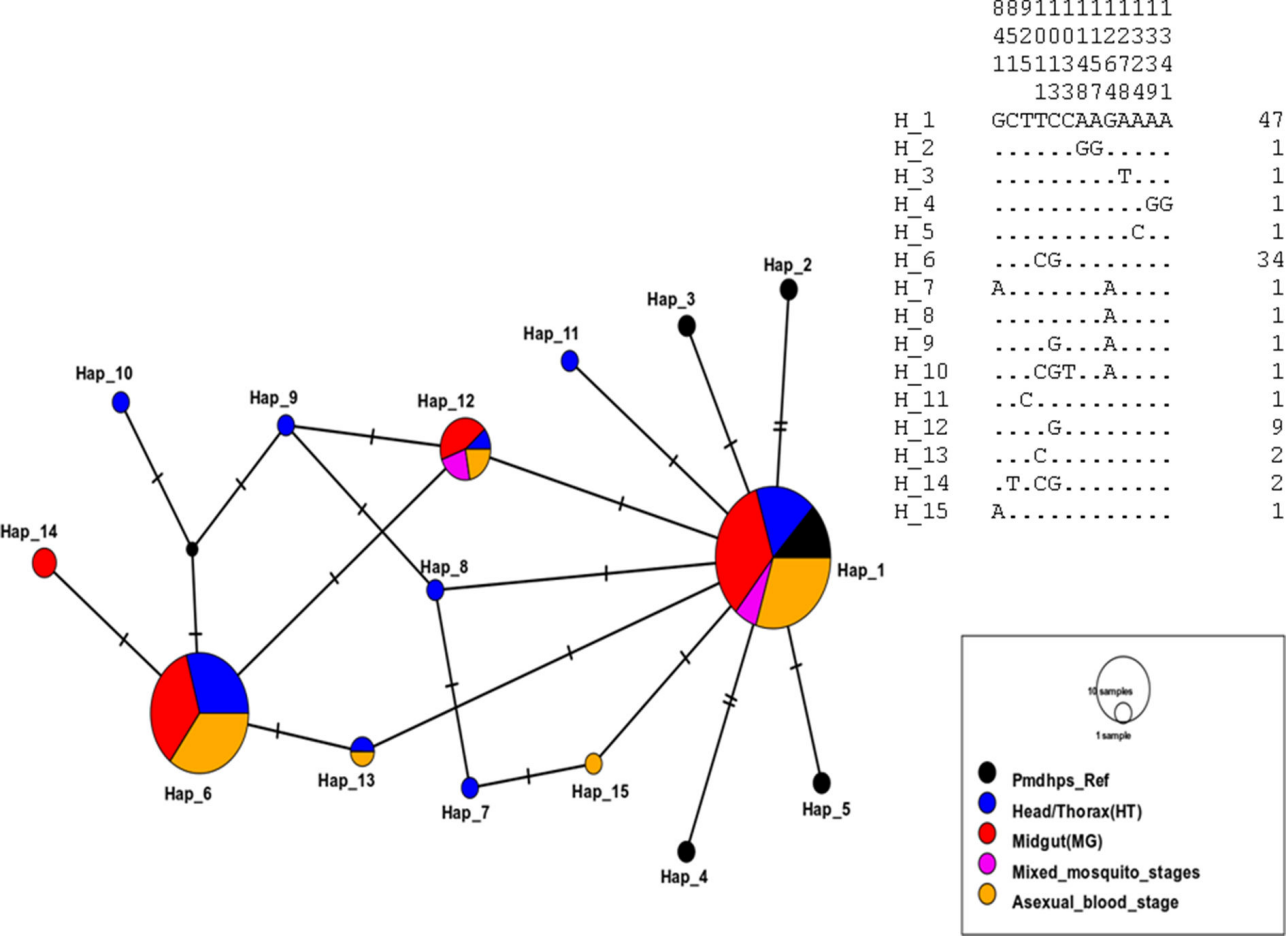
Haplotype structure of the sequenced *Pmdhps* isolates identifying multiple mutant parasite lineages.

Similarly, a 960-bp partial *Pmdhps* fragment was amplified (codons 265 to 554) from *P. malariae*-infected *Anopheles* mosquitoes and human samples, and sequencing was done for a total of 94 isolates (including 25 H/T stage, 34 midgut stage, 5 mixed mosquito stages, and 30 asexual blood stage) (Table S4). Sequence analysis revealed 872 nucleotide sites containing 07 variables (polymorphic) and 865 monomorphic positions (Table S9). The polymorphic sites comprised 01 synonymous and 06 non-synonymous polymorphisms in the amino acid coding sequence including major [A437**G** (^GCT^1311**^GGT^**) and S^436^**S** (^TCT^1310**^TCC^**)] and minority [D^380^**N**, (^GAT^1138**^AAT^**), S^383^**F** (^TCT^1148**^TTT^**), F^408^**L** (^TTT^1222**^CTT^**), P^444^**S** (^CCT^1330**^TCT^**), and E^521^**K** (^GAA^1330**^AAA^**)] types. The six amino acids in the *Pmdhps* analogous to residues linked with sulfadoxine resistance in *Pfdhps* backbone were all wild type (S^436^, K^540^, A^581^, and A^613^) except the mutant A^437^G (Fig. 3 and 4 & Table S4). The overall frequency rates of the predominant S^436^**S** and A^437^**G** alleles were 41.5% (39) and 50% (94), respectively, partitioned into H/T stage [^436^**S**: 30.8% (12) and ^437^**G**: 27.7% (47)], midgut stage [^436^**S**: 35.9% (14) and ^437^**G**: 38.3% (18)], mixed mosquito forms [^436^**S**: 0% and ^437^**G**: 4.3% (2)], and blood stage [^436^**S**: 33.3% (13) and ^437^**G**: 29.8% (14)]. Both the S^436^**S** and A^437^**G** alleles documented slightly similar frequencies across the H/T, midgut, and human blood stages. Similarly, no difference in the mixed (heterozygous) A^437^G polymorphism was observed (H/T: 13.6%, midgut: 11.7%, and human blood stage: 13.3%). Regarding the minority alleles, ^380^**N** (1.1%, 1/94), ^408^**L** (1.1%, 1/94), ^444^**S** (1.1%, 1/94), and ^521^**K** (4.3%, 4/94) were found in the H/T stage while ^383^**F** (2.1%, 2/94) was observed in the midgut stage. The double mutant **S^436^G^437^** was found at an overall frequency of 39.4% (37/94) and categorized into the H/T stage (29.7%, 11/37), midgut stage (37.8%, 14/37), and asexual blood stage (32.4%, 12/37) (Table S5). Similarly, the singleton A^437^**G** mutant type occurred at 8.5% (8/94) including H/T stage (12.5%, 1/8), midgut stage (37.5%, 3/8), mixed stage (25%, 2/8), and blood-stage (25%, 2/8). The wild-type population was at 44.7% (42/94). Genetic diversity deduces the circulation of seven haplotypes from the 94 indigenous sequences with a diversity score of 0.675 and a Tajima value of −0.28239 though not statistically significant (*P* > 0.10) (Table S9). The number of haplotypes was higher at the H/T stage (*n* = 9) than the midgut (*n* = 4) and asexual blood stages (*n* = 5). Evolutionary analysis of the multiple aligned nucleotide sequences (94 natural *Pmdhps* isolates + 10 GenBank-retrieved Thailand sequences) by the maximum-likelihood method and HKY model reveals three major phylogenetic clusters with the wild type uniquely pairing with the Thailand isolates and a segregating mutant population forming distinct assemblages (Fig. 9). Haplotype network construction with only the 94 sequence isolates shows seven haplotypes with an increment to 15 haplotypes upon combining the 10 Thailand sequences (Fig. 10). The wild type aligned with some of the Thailand sets (H1; *n* = 47) while the mutant isolates formed H6 (*n* = 34). Minority haplotypes constituted single Thailand sequences (H2–H5) and discrete units of the mutant field isolates (H7–H15) (Fig. 10). Individual analysis of H/T and midgut stage sequence clusters of *Pfdhfr*, *Pfdhps*, *Pmdhfr*, and *Pmdhps* reveals a significant proportion of shared haplotypes between the mosquito stages and asexual blood forms (Table S5).

## DISCUSSION

Accurate knowledge of drug resistance epidemiology is fundamental for malaria control and elimination. This becomes important as efforts to control malaria are encountering hurdles arising from the silent burden of asymptomatic infection (17) and the emergence of drug resistance (18). Molecular detection methods such as PCR have illuminated fine insights into parasite species composition, revealing the hidden burden of *P. malariae* which often presents as asymptomatic sub-microscopic parasitemia (19). Here, we report evidence of the increasing circulation of *P. malariae* as a significant cause of malaria in Cameroon often in mixed infection with *P. falciparum*, consistent with previous data documenting a high proportion of *P. malariae* infection in mosquitoes (11). This high *P. malariae* prevalence can be attributed to expanded access to anti-malarial interventions mainly directed to the treatment of *P. falciparum* infection while indirectly favoring the silent rise of the former and selecting for mutations in drug-resistant backbones. Indeed, the adaptive survival of *P. malariae* is sustained by a 72-hour life cycle characterized by waves of slow multiplication in infected erythrocytes before eventual lysis. Despite the high *Pf/Pm* co-infection prevalence, there was a reduction in *P. malariae* parasite density compared with *P. falciparum*. Inter-parasite species competition could be responsible for the comparatively low *P. malariae* parasitemic biomass as observed in sympatric infection. The superiority of *P. falciparum* in Cameroon still poses a challenge to the national malaria control program as the deployment of therapeutic interventions is in parallel favoring the emergence and spread of drug resistance. In addition, the high frequency of PCR-identified *P. falciparum* not detected by the conventional RDT poses a major concern particularly with the paucity of data on histidine-rich protein-2 gene deletion in Cameroon. Such *P. falciparum* diagnostic “refugia” phenotypes may compromise the performance and shelf-life of RDTs in malaria detection accounting for the substantial false negatives.

On the other hand, the malaria cycle involves *Plasmodium* parasites encountering dramatic environmental changes during transition between the mosquito and human hosts, which could impact fitness, population size, allele composition, and differential transmissibility of variants (20). Indeed, anti-malarial drugs and immune response in humans as well as population bottlenecks during ookinete migration across the midgut barrier and sporozoite invasion of the salivary gland in the mosquito definitive host can shape the overall landscape of parasite diversity and drug resistance allele frequency. In this regard, data generated from this study show the presence of mutations in the *Pfdhfr/Pfdhps* and *Pmdhfr/Pmdhps* gene backbones suggesting a directional selection of resistant parasite sub-populations by continuous SP drug pressure in the human host that are transmitted by mosquitoes (21). These mutations drive the evolution of SP resistance that led to the withdrawal of the drug as a monotherapy option against malaria (22). While the current SP formulation combines a partner drug, the persistent drug pressure still selects for drug resistance phenotypes that may culminate in the loss of drug efficacy for malaria control. This is supported by the saturation of the *Pfdhfr* **IRN** triple-mutant alleles in synergy with a high frequency of the *Pfdhps* A437**G** mutation in both host systems suggesting that resistance alleles to pyrimethamine and sulfadoxine are increasingly selected in the field (23, 24). This is of major concern since the *Pfdhps* 437**G** marker is associated with partial parasite resistance to sulfa drugs. The *Pfdhps* K540E mutation linked with full resistance was identified only in a single isolate, confirming the low prevalence of this allele in Cameroon similar to previous studies (23, 24).

Moreover, the novel *Pfdhps* I^431^**V** allele previously detected in Nigeria and observed to be spreading across Central Africa (25, 26) including Chad and the Democratic Republic of Congo was identified in this study at a frequency of 27.1%. This allele exhibited similar frequency (30.8%) in the H/T and human stages although lower in the midgut stage (18.1%), possibly because of sporozoite population expansion in the salivary gland from ruptured oocyst that successfully traversed the midgut barrier (20). Molecular docking evidence from Oguike et al. (27) showed that the I^431^**V** mutation enhances resistance to SP by lowering the binding affinity of the drug to the *dhps* enzyme target. Although the effect of the *Pfdhps*-431V mutation on SP resistance is not yet known, the progressive increase of this mutant allele is of prime concern to regularly monitor its impact on the effectiveness of SP-based IPT programs in Cameroon. Hence, the increasing frequency of this I^431^**V** co-existing with the A437**G** among heterogeneous parasite populations raises an alarm on the long-term therapeutic longevity of SP in Cameroon.

Despite this observation, the extensive use of antibiotics such as Cotrimoxazole (containing trimethoprim and sulfamethoxazole ingredients) known to select for *dhfr*/*dhps* mutant alleles could also be directly contributing to resistance expansion. Nonetheless, molecular surveillance data from this study highlight that routine implementation of SP as prophylaxis to prevent *P. falciparum* infection in pregnancy or in combination with amodiaquine (SP + AQ) for SMC programs in children in Cameroon is still effective despite the presence of mutations.

Furthermore, a comparison of polymorphism frequency in the genes mediating anti-folate sensitivity shows that *P. malariae* has encountered a relatively lower impact of SP selection pressure compared with *P. falciparum* even though both parasite species have been exposed to the same drug treatment over decades and this can be linked to the multiplication cycle of the former in infected erythrocytes. The present study provides the first genetic epidemiological data of *P. malariae* in Cameroon by characterizing the polymorphism profile in known SP drug resistance markers from the human intermediate and the mosquito definitive host systems of the parasite life cycle. The high frequency of the double **L^57^R^58^**-mutant haplotypes in the *Pmdhfr* backbone suggests a strong selection force imposed by pyrimethamine that has favored the emergence of these polymorphisms. The presence of heterozygous mixed alleles in the *Pmdhfr* asexual blood stage may suggest super-infection by distinct parasite genotypes arising from multiple infectious bites. Similar to *P. falciparum*, *P. malariae* is considered to acquire resistance to pyrimethamine more rapidly than sulfadoxine and this explains the reason for the high frequency of multiple polymorphisms in the *dhfr* than *dhps* backbones, consistent with previous studies (28, 29). The low frequency of the *Pmdhfr* S^114^N polymorphism in humans and the observed distant clustering pattern of this polymorphism with Thailand isolates may suggest the local independent emergence of this allele in Cameroon. In addition, a high proportion of the *Pmdhfr* isolates harboring mutations at amino acids S^58^R was observed alongside a low frequency of the S^114^N that corresponds to generic codon positions C^59^R and S^108^N in *Pfdhfr*, respectively. Sequential accumulation of these mutations in the *Pmdhfr* backbone could have probably originated from the C^59^R ancestral residue as inferred from its high frequency. While it has been demonstrated through phenotypic studies that the ^108^**N** and ^59^**R** primarily confer pyrimethamine resistance in *P. falciparum*, no data exist on whether the equivalent mutations are linked to pyrimethamine resistance in *P. malariae*.

Akin to *P. falciparum*, the *Pmdhps* sequence isolates reveal a high frequency of the dominant A^437^**G** allele in the gene backbone. This may suggest that the absence of key mutations in the sulfadoxine resistance-conferring *Pmdhps* gene could be attributed to detrimental fitness costs that may impact parasite survival and transmission, particularly in mixed species infection. Phylogenetic computation of the local *Pmdhfr* and *Pmdhps* sequences separated the field mutant sequences and the Thailand isolates as distinct clades suggesting divergent parasite populations further highlighting the possibility of the *de novo* emergence of these mutations.

Consistent with previous studies by reference (30, 31), comparative assessment of *P. falciparum* and *P. malariae dhfr* parasite populations reveals similar profiles of the mutant allele frequency and diversity between intra- (within-mosquito compartments) and inter- (between humans and mosquito) host systems. However, a contrast exists for the *dhps* 431V and 436A alleles with higher frequencies observed in the mosquito H/T and human stages than the midgut stage. This discrepancy could be linked to continuous pressure imposed by decades of SP use that is otherwise driving the adaptation and efficient transmission capacity of parasites harboring dominant *dhfr* mutant alleles with minimal fitness, thus, ensuring spread of the resistant variants across the definitive (mosquito) and intermediate (human) hosts. This study underscores the heterogeneity in transmission of different markers of anti-malarial drug resistance; further emphasizing that not all mutations are equal in terms of transmission-carrying capacity by local mosquito populations.

Despite the detection of mutations in these key parasite genes, current research to understand the treatment susceptibility outcome of *P. malariae* is limited by the difficulty in conducting *in vivo* therapeutic efficacy studies (TES) and the challenges of *in vitro* parasite culture (28). However, recently established *ex vivo* culture has facilitated conventional anti-malarial susceptibility evaluation (32) and the possibility of validating cross-species molecular markers which would be fundamental for future studies on this neglected parasite. Further studies employing microsatellite markers will be critical in identifying both the local geographical origin of the mutations as well as uncovering the population structure of *P. malariae* isolates in mixed infections and between the two host systems. The experimental infection system exploiting natural *P. falciparum*/*P. malariae* parasites in wild *Anopheles* vectors will shed insights on the role of mosquitoes in drug resistance genotype transmission. The limitation of the current study arises from the aspect that adult mosquitoes were randomly collected in households, thereby introducing a sampling bias since multiple blood feeding is characteristic of natural mosquito populations. Thus, parasite typing from the mosquito abdomen (midgut compartment) does not differentiate between asexual stages (trophozoites, schizonts) in the blood bolus and oocyst infection particularly over successive infectious meals. Secondly, the mosquitoes and human blood samples were not collected from the same households and sampling was done at different time periods. This hindered a real-time pair-wise comparison of the allelic frequency of molecular markers between the two host systems. Thirdly, Sanger sequencing is inferior by the inability to detect polymorphisms at minor frequencies especially in high transmission areas where super-infection is common. However, this can be resolved by the next-generation sequencing (NGS) technique that detects novel mutations and a minority allele variant in mixed infections, permitting allele frequency quantification in heterozygous genotypes. Nevertheless, this study provides relevant data intersecting the human-to-mosquito phenotypic attributes of drug resistance transmission, revealing that *Anopheles* mosquitoes contribute to maintaining the circulation and differential transmission of mutant variants in nature on a background of optimal parasite drug selection pressure in the human host. In conclusion, this study reveals a high prevalence of asymptomatic *P. falciparum* and *P. malariae* parasites harboring multiple drug resistance-associated mutations. Despite this observation, the low frequency of parasites with the quintuple dhps540E allele suggests the continuous efficacy of SP as a chemoprophylaxis regimen in Cameroon. Therapeutic efficacy studies accompanied by molecular analysis must be routinely implemented to monitor the clinical efficacy of SP and the parasitological impact on the emergence of resistant parasites for guiding effective anti-malaria policy

## MATERIALS AND METHODS

### Adult mosquito collection across Cameroon

Indoor-resting adult *Anopheles* mosquitoes were collected across 09 localities in Cameroon representing three major bio-ecological zones mainly equatorial (Bankeng, Bonaberi, Elende, Elon, Obout, Mangoum), Sudano-Guinean (Mibellon), and Sahel (Gounougou and Simatou) regions as previously published (11). The mosquitoes were identified morphologically using published keys (33), and sibling speciation of the *An. gambiae* complex and *An. funestus* group was done by molecular typing according to established protocols (34, 35).

### Dry blood spot collection from the human population and malaria parasite determination

Demographic data were collected from each participant using a questionnaire with capillary blood collected from each participant aged between 2 years and 50^+^ years in Mibellon (September 2020) (36) and Elende (August 2021). In Gounougou (March 2021), only the 3 to 15 years age group was recruited for finger-prick blood sampling as previously published (37). A community-based cross-sectional design was employed to actively recruit participants. Inclusion criteria comprised of individuals willing to participate and signed an informed consent form including approval from parents/guardian for children ≤ 18 years and residents in the village for >1 year. Exclusion criteria included non-consenting individuals, signs of chronic illness, and severe anemia (Hb level < 5 g/dL). Enrollment consists of assigning an identification code to each participant, and data collected were entered in a database. Temperature, weight, and height were collected for each participant. The CareStart RDT and Giemsa-based microscopic examination of blood films were used to determine the parasitological prevalence of malaria following standard protocols (38). Briefly, air-dried thin blood film was fixed in 75% methanol, and both thick and thin blood films were stained using 10% Giemsa solution for 20 min. The slides were then microscopically examined for the presence of malaria parasites. Malaria parasites were enumerated against 200 white blood cells (WBC) (or 500 WBCs in the case of very low parasitemia) in thick blood films. Parasite density was expressed as the number of parasites per microliter (μL) of blood, assuming a WBC count of 8,000 leucocytes per μL of blood. Parasitemia was categorized as low (<1,000 parasites/μL blood), moderate (10,00–4,999 parasites/μL blood), high (5,000–99,999 parasites/μL blood), and hyperparasitemia (≥100,000 µL) (39).

### DNA extraction and *Plasmodium* molecular species identification

The abdomen (Abd) and head/thorax (H/T) of a subset of female *Anopheles* mosquitoes were partitioned to discriminate between midgut (denoting the non-committed blood bolus asexual, zygote, and/or oocyst stages) and salivary gland (indicating sporozoite stage) infection. Extraction of genomic DNA from the H/T and Abd of individual dissected female mosquitoes from all the localities (except Obout for which whole mosquito samples were used) was done using the LIVAK method (40). Molecular identification was performed to discriminate against *An. funestus* siblings and the *An. gambiae* s.l species complex (41). Furthermore, the TaqMan assay employing the real-time PCR MX 3005 (Agilent, Santa Clara, CA, USA) system was used to detect *Plasmodium* infection in DNA extracts of wild *Anopheles* mosquito populations as previously published (11). In contrast, Whatman paper dry blood spots (DBS) were obtained from each human participant for genomic DNA extraction and molecular analysis of *Plasmodium* species composition. Genomic DNA was extracted from two dried blood spots on Whatman paper using chelex-100 (42) .

*Plasmodium* species detection targeting the 18S rRNA was done using a modified version of the nested PCR method as previously described (43, 44). The first round of amplification consists of a mix of genus-specific primers rPLU-5 (0.51 µL) and rPLU-6 (0.51 µL), with 11.62 µL of sterile water, 1.5 µL of 10× Kapa Taq buffer, 1.875 µL BSA, 0.12 µL of 25 mM dNTP, 0.75 µL of 25 mM MgCl_2_, 0.12 µL of Kapa Taq polymerase (BioLabs Inc.), and 5 µL of genomic DNA in a final volume of 20 µL. The second round of amplification comprised the same components except for 5 µL of the first PCR products and 10 µM of 0.51 µL each of *Plasmodium* species*-*specific primers (rFAL1 + rFAL2, rMAL1 + rMAL2, rOVAL1 + rOVAL2, and rVIV1 + rVIV2). The nested PCR products were separated on a 2% agarose gel to identify *P. falciparum* and *P. malariae* species.

### *P. falciparum* and *P. malariae dhfr* and *dhps* PCR gene amplification

The samples positive for *P. falciparum* and *P. malariae* from infected mosquitoes and human samples were each subjected to separate *dhfr* (28, 45) and *dhps* (29) nested PCR gene amplification. A 20-µL primary PCR volume was prepared for each gene amplification constituting 5 µL of the genomic DNA extract, 0.51 µL each outer forward and reverse primers, 0.12 µL each kappa Taq enzyme (Kappa Biosystems, Wilmington, MA, USA), 1.875 µL BSA and dNTP mix, 0.75 µL MgCl_2_, 1.5 µL kappa Taq buffer, and 11.62 µL distilled water. Similarly, the nested PCR followed the same master mix composition as the primary except that inner forward and reverse primers were used. After amplification, the nested PCR products of both genes were loaded on 2% agarose gel to reveal appropriate band sizes. Exo-SAP purification was done on randomly selected amplicons and sequenced commercially (Microsynth Seqlab GmbH, Germany) using the Sanger method.

### Data analysis on malaria prevalence and associated indicators

Data collected were entered in MS Excel, imported into, and analyzed using GraphPad Prism V8 (GraphPad Software, La Jolla, California USA). Participants were categorized into <5 years, 5–15 years, 16–30 years, and >30 years age groups. Continuous variables were summarized into means and standard deviations (SD), and categorical variables reported as frequencies and percentages were used to evaluate the descriptive statistics. The differences in proportions were evaluated using Pearson’s χ^2^. The level of agreement between parasite prevalence estimates determined by RDT and microscopy was inferred using Cohen’s kappa test. Significant levels were measured at a 95% confidence interval with statistically acceptable differences set at *P* < 0.05.

### Sequence analysis and genetic phylogeny

Sequence data FASTA files from the *dhfr* and *dhps* genes of *P. falciparum* and *P. malariae* were base called using BioEdit software and then submitted to the standard nucleotide basic local alignment search tool (BLAST) database search program to confirm the sequence identities. The *P. falciparum* sequences were then aligned against the 3D7 wild-type *Pfdhfr* (PF3D7_0417200) and *Pfdhps* (PF3D7_0810800) sequences for reference obtained from PlasmoDB (www.Plasmodb.org). The *P. malariae dhfr* sequences were aligned with the Thailand isolates (Accession numbers: AY846633, AY846634, EF188271, EF188273, EF188274, and KX672034) as reference (46). Similarly, the *Pmdhps* isolates were matched with Thailand GenBank samples (Accession numbers: KJ400020, KJ400021, KJ400022, KJ400023, KJ400024, KJ400025, KX672040, KX672041, KX672042, and KX672043) (46). Particularly, the *Pfdhfr* and *Pfdhps* gene sequences were scanned for previously identified point mutations in resistance-associated codons. Identified SNPs related to drug resistance molecular markers were classified as either wild, mutant, or mixed. However, mixed genotypes were considered as mutant when constructing haplotypes. The haplotype diversity (the number of two random strains within the population having different haplotypes) of Pfdhfr/Pfdhps and Pmdhfr/Pmdhps in each sequenced field isolate was determined by exploring the variants in the coding region of the gene. DnaSP software (version 6.10.01) was used to interrogate the genetic diversity metrics of the parasite population per the host system and locality. Genealogical relationship between individual parasites and haplotypes was generated by MEGA X (47), Templeton, Crandall, and Sing (TCS) haplotype network and PopArt software (48).

## Data Availability

All data generated are included in the article and the supplementary file. Nucleotide sequences have been uploaded to the NCBI under GenBank accession numbers OQ774115 - OQ774779.

## SUPPLEMENTAL MATERIAL

Tables S1 to S9 aac.00588-23-s0001.docx Supplementary tables showing allele frequency of molecular markers and genetic diversity statistics

ASM does not own the copyrights to Supplemental Material that may be linked to, or accessed through, an article. The authors have granted ASM a non-exclusive, world-wide license to publish the Supplemental Material files. Please contact the corresponding author directly for reuse.
