# Supplementary material for: Geographical emergence of sulfadoxine-pyrimethamine drug resistance-associated P. falciparum and P. malariae alleles in co-existing Anopheles mosquito and asymptomatic human populations across Cameroon
Source: Antimicrob Agents Chemother. 2023 Nov 10;67(12):e00588-23. doi: 10.1128/aac.00588-23 (PMC10720508; doi:10.1128/aac.00588-23)
Supplement: Tables S1 to S9 — Supplementary tables showing allele frequency of molecular markers and genetic diversity statistics [file aac.00588-23-s0001.docx]

**Supplementary Table 1:** Allelic frequency of polymorphisms in the *Pfdhfr* backbone

| ***Pfdhfr*** | | | | | | | | | | | | | |
| --- | --- | --- | --- | --- | --- | --- | --- | --- | --- | --- | --- | --- | --- |
| **H/T stage** | | |  | | **Allelic frequency; n (%)** | | | | | | | | |
|  |  |  |  |  | **N51I** | | | **C59R** | | | **S108N** | | |
| **Locality** | ***Anopheles*** | | **N** | | **Wild**  **(N51)** | **Mutant**  **(51I)** | **Mixed**  **(51N/I)** | **Wild**  **(C59)** | **Mutant**  **(59R)** | **Mixed**  **(59C/R)** | **Wild**  **(S108)** | **Mutant**  **(108N)** | **Mixed**  **(108S/N)** |
| Bankeng | AGAM |  | 17 | | 0 (0.0) | 16 (94.1) | 1 (5.9) | 3 (17.6) | 12 (70.6) | 2 (11.8) | 0 (0.0) | 17 (100) | 0 (0.0) |
| Elende | AFUN |  | 13 | | 0 (0.0) | 13 (100) | 0 (0.0) | 0 (0.0) | 13 (100) | 0 (0.0) | 0 (0.0) | 13 (100) | 0 (0.0) |
| Elon | AFUN |  | 5 | | 0 (0.0) | 5 (100) | 0 (0.0) | 0 (0.0) | 5 (100) | 0 (0.0) | 0 (0.0) | 5 (100) | 0 (0.0) |
| Gounougou | ACOL |  | 9 | | 0 (0.0) | 8 (88.9) | 1 (11.1) | 2 (22.2) | 6 (66.7) | 1 (11.1) | 0 (0.0) | 9 (100) | 0 (0.0) |
| Mibellon | AFUN |  | 15 | | 0 (0.0) | 13 (86.7) | 2 (13.3) | 1 (6.7) | 12 (80) | 2 (13.3) | 1 (6.7) | 14 (93.3) | 0 (0.0) |
| Simatou | ACOL |  | 11 | | 0 (0.0) | 9 (81.8) | 2 (18.2) | 0 (0.0) | 11 (100) | 0 (0.0) | 0 (0.0) | 11 (100) | 0 (0.0) |
| **Total** | / |  | **70** | | **0 (0.0)** | **64 (91.4)** | **6 (8.6)** | **6 (8.6)** | **59 (84.3)** | **5 (7.1)** | **1 (1.4)** | **69 (98.6)** | **0 (0.0)** |
| **Midgut stage** | |  | | | | | | | | | | | |
| Bonaberi | ACOL |  | | 2 | 0 | 2 (100) | 0 (0.0) | 0 (0.0) | 2 (100) | 0 (0.0) | 0 (0.0) | 2 (100) | 0 (0.0) |
| Elende | AFUN |  | | 15 | 0 | 15 (100) | 0 (0.0) | 0 (0.0) | 15 (100) | 0 (0.0) | 0 (0.0) | 15 (100) | 0 (0.0) |
| Elon | AFUN |  | | 15 | 3 (20) | 12 (80) | 0 (0.0) | 3 (20) | 12 (80) | 0 (0.0) | 3 (20) | 12 (80) | 0 (0.0) |
| Gounougou | ACOL |  | | 13 | 0 | 13 (100) | 0 (0.0) | 0 (0.0) | 13 (100) | 0 (0.0) | 0 (0.0) | 13 (100) | 0 (0.0) |
| Mangoum | AGAM |  | | 15 | 0 | 15 (100) | 0 (0.0) | 1 (6.7) | 14 (93.3) | 0 (0.0) | 0 (0.0) | 15 (100) | 0 (0.0) |
| Mibellon | AFUN |  | | 17 | 0 | 17 (100) | 0 (0.0) | 0 (0.0) | 17 (100) | 0 (0.0) | 0 (0.0) | 17 (100) | 0 (0.0) |
| Simatou | ACOL |  | | 12 | 0 | 12 (100) | 0 (0.0) | 0 (0.0) | 12 (100) | 0 (0.0) | 0 (0.0) | 12 (100) | 0 (0.0) |
| **Total** | / |  | | **89** | **3 (3.4)** | **86 (96.6)** | **0 (0.0)** | **4 (4.5)** | **85 (95.5)** | **0 (0.0)** | **3 (3.4)** | **86 (96.6)** | **0 (0.0)** |
| **Mixed stages** | |  | | | | | | | | | | | |
| Obout | AFUN |  | | **17** | 0 (0.0) | 15 (88.2) | 2 (11.8) | 0 (0.0) | 17 (100) | 0 (0.0) | 0 (0.0) | 17 (100) | 0 (0.0) |
| **Blood stage** | |  | | | | | | | | | | | |
| Elende | **Human** |  | | 20 | 2 (10) | 17 (85) | 1 (5) | 0 (0.0) | 20 (100) | 0 (0.0) | 0 (0.0) | 20 (100) | 0 (0.0) |
| Mibellon |  |  |  | 20 | 0 (0.0) | 20 (100) | 0 (0.0) | 0 (0.0) | 20 (100) | 0 (0.0) | 0 (0.0) | 20 (100) | 0 (0.0) |
| Gounougou |  |  |  | 18 | 1 (5.6) | 16 (88.8) | 1 (5.6) | 0 (0.0) | 18 (100) | 0 (0.0) | 0 (0.0) | 18 (100) | 0 (0.0) |
| **Total** |  |  |  | **58** | **3 (5.2)** | **53 (91.4)** | **2 (3.4)** | **0 (0.0)** | **58 (100)** | **0 (0.0)** | **0 (0.0)** | **58 (100)** | **0 (0.0)** |

**Supplementary Table 2:** Allelic frequency of key polymorphisms in the *Pfdhps* backbone

| ***Pfdhps*** | | | | | | | | | | | | | |
| --- | --- | --- | --- | --- | --- | --- | --- | --- | --- | --- | --- | --- | --- |
| **H/T stage** | | |  | **Allelic frequency; n (%)** | | | | | | | | | |
|  |  |  |  | **I431V** | | | | **S436A** | | | **A437G** | | |
| **Locality** | ***Anopheles*** | | **N** | **Wild**  **(I431)** | | **Mutant**  **(431V)** | **Mixed**  **(431N/I)** | **Wild**  **(S436)** | **Mutant**  **(436A)** | **Mixed**  **(436S/A)** | **Wild**  **(A437)** | **Mutant**  **(437G)** | **Mixed**  **(437A/G)** |
| Bankeng | AGAM |  | 14 | 9 (64.3) | | 5 (35.7) | 0 (0.0) | 7 (50) | 7 (50) | 0 (0.0) | 0 (0.0) | 14 (100) | 0 (0.0) |
| Elende | AFUN |  | 12 | 9 (75) | | 3 (33.3) | 0 (0.0) | 7 (58.3) | 3 (25) | 2 (16.7) | 0 (0.0) | 12 (100) | 0 (0.0) |
| Elon | AFUN |  | 5 | 4 (80) | | 1 (20) | 0 (0.0) | 4 (80) | 1 (20) | 0 (0.0) | 0 (0.0) | 5 (100) | 0 (0.0) |
| Gounougou | ACOL |  | 9 | 5 (55.6) | | 4 (44.4) | 0 (0.0) | 3 (33.3) | 5 (55.6) | 1 (11.1) | 1 (11.1) | 7 (77.8) | 1 (11.1) |
| Mibellon | AFUN |  | 15 | 12 (80) | | 2 (20) | 1 (6.7) | 11 (73.3) | 4 (26.7) | 0 (0.0) | 2 (13.3) | 13 (86.7) | 0 (0.0) |
| Simatou | ACOL |  | 10 | 5 (50) | | 5 (50) | 0 (0.0) | 3 (30) | 5 (50) | 2 (20) | 2 (20) | 8 (80) | 0 (0.0) |
| **Total** | **/** |  | **65** | **44 (67.7)** | | **20 (30.8)** | **1 (1.5)** | **35 (53.8)** | **25 (38.5)** | **5 (7.7)** | **5 (7.7)** | **59 (90.8)** | **1 (1.5)** |
| **Midgut stage** | | | | |  | | | | | | | | |
| Elende | AFUN |  | 15 | 12 (80) | | 2 (13.3) | 1 (6.7) | 10 (66.7) | 2 (13.3) | 3 (20) | 0 (0.0) | 12 (80) | 3 (20) |
| Elon | AFUN |  | 14 | 13 (92.9) | | 1 (7.1) | 0 (0.0) | 13 (92.9) | 1 (7.1) | 0 (0.0) | 1 (7.1) | 13 (92.9) | 0 (0.0) |
| Gounougou | ACOL |  | 12 | 12 (100) | | 0 (0.0) | 0 (0.0) | 11 (91.7) | 1 (8.3) | 0 (0.0) | 0 (0.0) | 12 (100) | 0 (0.0) |
| Mangoum | AGAM |  | 14 | 5 (35.7) | | 7 (50) | 2 (14.3) | 3 (21.4) | 3 (21.4) | 8 (57.1) | 0 (0.0) | 14 (100) | 0 (0.0) |
| Mibellon | AFUN |  | 17 | 14 (82.4) | | 3 (17.6) | 0 (0.0) | 14 (82.4) | 1 (5.9) | 2 (11.8) | 0 (0.0) | 17 (100) | 0 (0.0) |
| Simatou | ACOL |  | 11 | 7 (63.6) | | 2 (18.2) | 2 (18.2) | 6 (54.5) | 3 (27.3) | 2 (18.2) | 0 (0.0) | 11 (100) | 0 (0.0) |
| **Total** | **/** |  | **83** | **63 (75.9)** | | **15 (18.1)** | **5 (6.0)** | **57 (68.7)** | **11 (13.3)** | **15 (18.1)** | **1 (1.2)** | **79 (95.2)** | **3 (3.6)** |
| **Mixed stages** | | | | |  | | | | | | | | |
| Obout | AFUN |  | **18** | 16 (88.9) | | 2 (11.1) | 0 (0.0) | 16 (88.9) | 2 (11.1) | 0 (0.0) | 0 (0.0) | 18 (100) | 0 (0.0) |
|  | | | | | | | | | | | | | |
| Elende | **Human** |  | 18 | 13 (72.2) | | 5 (27.8) | 0 (0.0) | 13 (72.2) | 4 (22.2) | 1 (5.6) | 1 (5.6) | 17 (94.4) | 0 (0.0) |
| Mibellon |  |  | 20 | 15 (75) | | 5 (25) | 0 (0.0) | 14 (70) | 6 (30) | 0 (0.0) | 3 (15) | 17 (85) | 0 (0.0) |
| Gounougou |  |  | 17 | 10 (58.8) | | 7 (41.2) | 0 (0.0) | 9 (52.9) | 8 (47.1) | 0 (0.0) | 4 (23.5) | 13 (76.5) | 0 (0.0) |
| Total |  |  | **55** | **38 (69.1)** | | **17 (30.9)** | **0 (0.0)** | **36 (65.5)** | **18 (32.7)** | **1 (1.8)** | **8 (14.5)** | **47 (85.5)** | **0 (0.0)** |

**Supplementary Table 3:** Polymorphism profile of the *Pmdhfr* backbone

| ***Pmdhfr*** | | | | | | | | | | | | |
| --- | --- | --- | --- | --- | --- | --- | --- | --- | --- | --- | --- | --- |
| **H/T stage** | | |  | **Allelic frequency; n (%)** | | | | | | | | |
|  |  |  |  | **F57L** | | | **S58R** | | | **S114N** | | |
| **Locality** | ***Anopheles*** | | **N** | **Wild (F57)** | **Mutant (57L)** | **Mixed (57F/L)** | **Wild (S58)** | **Mutant (58R)** | **Mixed (58S/R)** | **Wild (S114)** | **Mutant (114N)** | **Mixed (114S/N)** |
| Elende | AFUN |  | 06 | 1 (16.7) | 5 (83.3) | 0 (0.0) | 1 (16.7) | 5 (83.3) | 0 (0.0) | 0 (0.0) | 0 (0.0) | 0 (0.0) |
| Gounougou | ACOL |  | 05 | 2 (40) | 3 (60) | 0 (0.0) | 2 (40) | 3 (60) | 0 (0.0) | 0 (0.0) | 0 (0.0) | 0 (0.0) |
| Bankeng | AGAM |  | 04 | 0 (0.0) | 4 (100) | 0 (0.0) | 0 (0.0) | 4 (100) | 0 (0.) | 0 (0.0) | 0 (0.0) | 0 (0.0) |
| Mibellon | AFUN |  | 11 | 1 (9.1) | 10 (90.9) | 0 (0.0) | 1 (9.1) | 10 (90.9) | 0 (0.0) | 0 (0.0) | 0 (0.0) | 0 (0.0) |
| **Total** | / |  | **26** | **4 (15.4)** | **22 (84.6)** | **0 (0.0)** | **4 (15.4)** | **22 (84.6)** | **0 (0.0)** | **0 (0.0)** | **0 (0.0)** | **0 (0.0)** |
| **Midgut stage** | |  | | | | | | | | | | |
| Elon | AFUN |  | 5 | 2 (20) | 3 (80) | 0 (0.0) | 2 (20) | 3 (80) | 0 (0.0) | 0 (0.0) | 0 (0.0) | 0 (0.0) |
| Elende | AFUN |  | 10 | 0 (0.0) | 10 (100) | 0 (0.0) | 0 (0.0) | 10 (100) | 0 (0.0) | 0 (0.0) | 0 (0.0) | 0 (0.0) |
| Gounougou | ACOL |  | 6 | 2 (33.3) | 4 (66.7) | 0 (0.0) | 2 (33.3) | 4 (66.7) | 0 (0.0) | 0 (0.0) | 0 (0.0) | 0 (0.0) |
| Mangoum | AGAM |  | 3 | 0 (0.0) | 3 (100) | 0 (0.0) | 0 (0.0) | 3 (100) | 0 (0.0) | 0 (0.0) | 0 (0.0) | 0 (0.0) |
| Mibellon | AFUN |  | 12 | 2 (16.7) | 10 (83.3) | 0 (0.0) | 2 (16.7) | 10 (83.3) | 0 (0.0) | 0 (0.0) | 0 (0.0) | 0 (0.0) |
| Simatou | ACOL |  | 3 | 2 (66.7) | 1 (33.3) | 0 (0.0) | 2 (66.7) | 1 (33.3) | 0 (0.0) | 0 (0.0) | 0 (0.0) | 0 (0.0) |
| **Total** | **/** |  | **39** | **8 (20.5)** | **31 (79.5)** | **0 (0.0)** | **8 (20.5)** | **31 (79.5)** | **0 (0.0)** | **0 (0.0)** | **0 (0.0)** | **0 (0.0)** |
| **Mixed stages** | |  | | | | | | | | | | |
| Obout | AFUN |  | **9** | 0 (0.0) | 9 (100) | 0 (0.0) | 0 (0.0) | 9 (100) | 0 (0.0) | 0 (0.0) | 0 (0.0) | 0 (0.0) |
| **Blood stage** | | | | | | | | | | | | |
| Elende | **Human** |  | 15 | 3 (20) | 12 (80) | 0 (0.0) | 2 (13.3) | 13 (86.7) | 0 (0.0) | 13 (86.6) | 1 (6.7) | 1 (6.7) |
| Mibellon |  |  | 17 | 4 (23.5) | 10 (58.8) | 3 (17.7) | 0 (0.0) | 11 (64.7) | 6 (35.3) | 17 (100) | 0 (0.0) | 0 (0.0) |
| Gounougou |  |  | 10 | 2 (20) | 7 (70) | 1 (10) | 0 (0.0) | 9 (90) | 1 (10) | 9 (90) | 0 (0.0) | 1 (10) |
| Total |  |  | **42** | **9 (21.4)** | **29 (69.1)** | **4 (9.5)** | **2 (4.8)** | **33 (78.6)** | **7 (16.7)** | **39 (92.9)** | **1 (2.3)** | **2 (4.8)** |

**Supplementary Table 4:** Polymorphisms profile of the *Pmdhps* backbone

| ***Pmdhps*** | | | | | | | | | | | | | | |
| --- | --- | --- | --- | --- | --- | --- | --- | --- | --- | --- | --- | --- | --- | --- |
| **H/T stage** | | |  | **Allelic frequency; n (%)** | | | | | | | | | | |
|  |  |  |  | **A437G** | | | | **E521K** | | | | **Minor mutant allele** | | |
| **Locality** | ***Anopheles*** | | **N** | **Wild (A437)** | | **Mutant (437G)** | **Mixed (437A/G)** | **Wild (E521)** | | **Mutant (521K)** | **Mixed (521E/K)** | **D380N** | **F408L** | **P444S** |
| Elende | AFUN |  | 03 | 3 (100) | | 0 (0.0) | 0 (0.0) | 3 (100) | | 0 (0.0) | 0 (0.0) | 0 (0.0) | 0 (0.0) | 0 (0.0) |
| Gounougou | ACOL |  | 05 | 3 (60) | | 2 (40) | 0 (0.0) | 3 (60) | | 0 (0.0) | 2 (40) | 1 (20) | 0 (0.0) | 0 (0.0) |
| Bankeng | AGAM |  | 04 | 1(25) | | 2 (50) | 1 (25) | 2 (50) | | 0 (0.0) | 2 (50) | 0 (0.0) | 1 (25) | 1 (25) |
| Mibellon | AFUN |  | 10 | 5 (50) | | 3 (30) | 2 (20) | 10 (100) | | 0 (0.0) | 0 (0.0) | 0 (0.0) | 0 (0.0) | 0 (0.0) |
| **Total** | / |  | **22** | **12 (54.5)** | | **7 (31.8)** | **3 (13.6)** | **18 (81.8)** | | **0 (0.0)** | **4 (18.2)** | 1 (4.5) | 1 (4.5) | 1 (4.5) |
|  | |  | | | | | | | | | | | | |
| **Midgut stage** | |  | | **S383F** | | | | | **S435L** | | | **A437G** | | |
|  |  |  |  | **Wild**  **(S383)** | | **Mutant**  **(383F)** | **Mixed**  **(383S/F)** | **Wild**  **(S435)** | | **Mutant**  **(435L)** | **Mixed**  **(435S/L)** | **Wild (A437)** | **Mutant (437G)** | **Mixed (437A/G)** |
| Elon | AFUN |  | 5 | 5 (100) | | 0 (0.0) | 0 (0.0) | 5 (100) | | 0 (0.0) | 0 (0.0) | 3 (60) | 2 (40) | 0 (0.0) |
| Elende | AFUN |  | 10 | 8 (80) | | 2 (20) | 0 (0.0) | 2 (20) | | 0 (0.0) | 8 (80) | 3 (30) | 6 (60) | 1 (10) |
| Gounougou | ACOL |  | 5 | 5 (100) | | 0 (0.0) | 0 (0.0) | 5 (100) | | 0 (0.0) | 0 (0.0) | 3 (60) | 2 (40) | 0 (0.0) |
| Mangoum | AGAM |  | 3 | 3 (100) | | 0 (0.0) | 0 (0.0) | 3 (100) | | 0 (0.0) | 0 (0.0) | 0 (0.0) | 2 (66.7) | 1 (33.3) |
| Mibellon | AFUN |  | 11 | 11 (100) | | 0 (0.0) | 0 (0.0) | 11 (100) | | 0 (0.0) | 0 (0.0) | 7 (63.6) | 2 (18.2) | 2 (18.2) |
| **Total** | **/** |  | **34** | **32 (94.1)** | | **2 (5.9)** | **0 (0.0)** | **26 (76.5)** | | **0 (0.0)** | **8 (23.5)** | **16 (47.1)** | **14 (41.2)** | **4 (11.7)** |
| **Mixed stages** | |  | | | | | | | | | | | | |
| Obout | AFUN |  | 7 | 7 (100) | | 0 (0.0) | 0 (0.0) | 7 (100) | | 0 (0.0) | 0 (0.0) | 5 (71.4) | 0 (0.0) | 2 (28.6) |
|  | | | | | | | | | | | | | | |
| **Blood stage** | | | | | **A437G** | | | **E521K** | | | | **K540E** | | |
| Elende | **Human** |  | 10 | 3 (30) | | 5 (50) | 2 (20) | 0 (0.0) | | 0 (0.0) | 0 (0.0) | 0 (0.0) | 0 (0.0) | 0 (0.0) |
| Mibellon |  |  | 10 | 6 (60) | | 2 (20) | 2 (20) | 0 (0.0) | | 0 (0.0) | 0 (0.0) | 0 (0.0) | 0 (0.0) | 0 (0.0) |
| Gounougou |  |  | 10 | 7 (70) | | 3 (30) | 0 (0.0) | 0 (0.0) | | 0 (0.0) | 0 (0.0) | 0 (0.0) | 0 (0.0) | 0 (0.0) |
| Total |  |  | **30** | **16 (53.3)** | | **10 (33.3)** | **4 (13.3)** | **0 (0.0)** | | **0 (0.0)** | **0 (0.0)** | **0 (0.0)** | **0 (0.0)** | **0 (0.0)** |

**Supplementary Table 5:** Haplotype combination of *P. falciparum* and *P. malariae dhfr* and *dhps* alleles

| **Genotypes** | | | | **H/T stage** | | **Midgut stage** | **Mixed mosquito stage** | **Humans**  **(blood stage)** | **Total** |
| --- | --- | --- | --- | --- | --- | --- | --- | --- | --- |
| ***P. falciparum dhfr* and *dhps* wild type genotype; % (n)** | | | | | | | | | |
| Wild type dhfr | | A^16^N^51^C^59^S^108^I^164^ | | 0.0 (0/70) | | 3.4 (3/89) | 0.0 (0/17) | 0.0 (0/58) | 3.4 (3/234) |
| Wild type dhps | | I^431^S^436^A^437^K^540^A^581^A^613^ | | 3.1 (2/65) | | 1.2 (1/83) | 0.0 (0/18) | 1.8 (1/55) | 1.8 (4/221) |
| ***P. falciparum dhfr* and *dhps* mutant genotype frequency; % (n)** | | | | | | | | | |
| Singleton dhfr | | A^16^**I^51^**C^59^S^108^I^164^ | | 1.4 (1/70) | | 0.0 (0/89) | 0.0 (0/17) | 0.0 (0/58) | 0.4 (1/234) |
| Double dhfr | | A^16^**I^51^**C^59^**N^108^**I^164^ | | 7.1 (5/70) | | 1.1 (1/89) | 0.0 (0/17) | 0.0 (0/58) | 2.6 (6/234) |
|  |  | A^16^N^51^**R^59^N^108^**I^164^ | | 0.0 (0/70) | | 0.0 (0/89) | 0.0 (0/17) | 5.2 (3/58) | 1.3 (3/234) |
| Triple dhfr | | A^16^**I^51^R^59^N^108^**I^164^ | | 91.4 (64/70) | | 95.5 (85/89) | 100 (17/17) | 94.8 (55/58) | 94.4 (221/234) |
| Singleton dhps | | **V^431^**S^436^G^437^K^540^A^581^A^613^ | | 0.0 (0/65) | | 0.0 (0/83) | 0.0 (0/18) | 1.8 (1/55) | 0.5 (1/221) |
|  |  | I^431^**A^436^**G^437^K^540^A^581^A^613^ | | 6.2 (4/65) | | 2.4 (2/83) | 0.0 (0/18) | 5.5 (3/55) | 4.1 (9/221) |
|  |  | I^431^S^436^**G^437^**K^540^A^581^A^613^ | | 47.7 (31/65) | | 69.9 (58/83) | 88.9 (16/18) | 58.2 (32/55) | 61.9 (137/221) |
| Double dhps | | **V^431^A^436^**G^437^K^540^A^581^A^613^ | | 0.0 (0/65) | | 0.0 (0/83) | 0.0 (0/18) | 7.3 (4/55) | 1.8 (4/221) |
|  |  | I^431^**A^436^G^437^**K^540^A^581^A^613^ | | 10.8 (7/65) | | 2.4 (2/83) | 0.0 (0/18) | 5.5 (3/55) | 5.4 (12/221) |
|  |  | **V^431^**S^436^**G^437^**K^540^A^581^A^613^ | | 3.1 (2/65) | | 4.8 (4/83) | 0.0 (0/83) | 3.6 (2/55) | 3.6 (8/221) |
| Triple dhps | | **V^431^A^436^G^437^**K^540^A^581^A^613^ | | 29.2 (19/65) | | 19.3 (16/83) | 11.1 (2/18) | 16.4 (9/55) | 20.8 (46/221) |
| Quadruple dhps | | **V^431^A^436^G^437^E^540^**A^581^A^613^ | | 0.0 (0/65) | | 0.0 (0/83) | 0.0 (0/18) | 1.8 (1/55) | 0.5 (1/221) |
| **Combined** ***P. falciparum dhfr* + *dhps* mutant genotype frequency; % (n)** | | | | | | | | | |
| Wild type | | A^16^N^51^C^59^S^108^I^164^_ I^431^S^436^A^437^K^540^A^581^A^613^ | 0.0 (0/65) | | | 0.0 (0/83) | 0.0 (0/18) | 0.0 (0/55) | 0.0 (0/221) |
| Single mutant | | A^16^**I^51^**C^59^S^108^I^164^_ I^431^S^436^A^437^K^540^A^581^A^613^ | 0.0 (0/65) | | | 0.0 (0/83) | 0.0 (0/18) | 0.0 (0/55) | 0.0 (0/221) |
| Double mutant | | A^16^**I^51^**C^59^**N^108^**I^164^_ I^431^S^436^A^437^K^540^A^581^A^613^ | 0.0 (0/65) | | | 0.0 (0/83) | 0.0 (0/18) | 0.0 (0/55) | 0.0 (0/221) |
|  |  | A^16^N^51^**R^59^N^108^**I^164^_ I^431^S^436^A^437^K^540^A^581^A^613^ | 0.0 (0/65) | | | 0.0 (0/83) | 0.0 (0/18) | 0.0 (0/55) | 0.0 (0/221) |
| Triple mutant | | A^16^**I^51^**C^59^**N^108^**I^164^_ I^431^**A^436^**A^437^K^540^A^581^A^613^ | 0.0 (0/65) | | | 0.0 (0/83) | 0.0 (0/18) | 0.0 (0/55) | 0.0 (0/221) |
|  |  | A^16^**I^51^**C^59^**N^108^**I^164^_ **V^431^**S^436^A^437^K^540^A^581^A^613^ | 0.0 (0/65) | | | 0.0 (0/83) | 0.0 (0/18) | 0.0 (0/55) | 0.0 (0/221) |
|  |  | A^16^**I^51^R^59^N^108^**I^164^_ I^431^S^436^A^437^K^540^A^581^A^613^ | 3.1 (2/65) | | | 1.2 (1/83) | 0.0 (0/18) | 1.8 (1/55) | 1.8 (4/221) |
|  |  | A^16^N^51^**R^59^N^108^**I^164^_ I^431^S^436^**G^437^**K^540^A^581^A^613^ | 0.0 (0/65) | | | 0.0 (0/83) | 0.0 (0/18) | 3.6 (2/55) | 0.9 (2/221) |
| Quadruple mutant | | A^16^**I^51^R^59^N^108^**I^164^_ **V^431^**S^436^A^437^K^540^A^581^A^613^ | 0.0 (0/65) | | | 0.0 (0/83) | 0.0 (0/18) | 1.8 (1/55) | 0.5 (1/221) |
|  |  | A^16^**I^51^R^59^N^108^**I^164^_ I^431^**A^436^**A^437^K^540^A^581^A^613^ | 6.2 (4/65) | | | 3.6 (3/83) | 0.0 (0/18) | 3.6 (2/55) | 4.1 (9/221) |
|  |  | A^16^**I^51^**C^59^**N^108^**I^164^_ **V^431^A^436^**A^437^K^540^A^581^A^613^ | 0.0 (0/65) | | | 0.0 (0/83) | 0.0 (0/18) | 0.0 (0/55) | 0.0 (0/221) |
|  |  | A^16^N^51^**R^59^N^108^**I^164^_ **V^431^A^436^**A^437^K^540^A^581^A^613^ | 0.0 (0/65) | | | 0.0 (0/83) | 0.0 (0/18) | 0.0 (0/55) | 0.0 (0/221) |
| Quintuple mutant | | A^16^**I^51^R^59^N^108^**I^164^_ **V^431^A^436^**A^437^K^540^A^581^A^613^ | 0.0 (0/65) | | | 0.0 (0/83) | 0.0 (0/18) | 7.3 (4/55) | 1.8 (4/221) |
|  |  | A^16^**I^51^R^59^N^108^**I^164^_ I^431^**A^436^G^437^**K^540^A^581^A^613^ | 9.2 (6/65) | | | 2.4 (2/83) | 0.0 (0/18) | 3.6 (2/55) | 4.5 (10/221) |
| Sextuple mutant | | A^16^**I^51^R^59^N^108^**I^164^_ **V^431^A^436^G^437^**K^540^A^581^A^613^ | 27.7 (18/65) | | | 19.3 (16/83) | 11.1 (2/18) | 16.4 (9/55) | 20.4 (45/221) |
| Septuplet mutant | | A^16^**I^51^R^59^N^108^**I^164^_ **V^431^A^436^G^437^E^540^**A^581^A^613^ | 0.0 (0/65) | | | 0.0 (0/83) | 0.0 (0/18) | 1.8 (1/55) | 0.5 (1/221) |
| ***P. malariae* *dhfr* and *dhps* wild type genotype frequency; % (n)** | | | | | | | | | |
| Wild type dhfr | N^50^K^55^F^57^S^58^S^59^S^114^F^168^I^170^ | | | 15.4 (4/26) | | 25.6 (10/39) | 0.0 (0/9) | 0.0 (0/42) | 12.1 (14/116) |
| Wild type dhps | S^436^A^437^K^540^A^581^A^613^ | | | 32 (8/25) | | 47.1 (16/34) | 60 (3/5) | 46.7 (14/30) | 43.6 (41/94) |
| Wild dhfr + dhps | N^50^K^55^F^57^S^58^S^59^S^114^F^168^I^170^_ S^436^A^437^K^540^A^581^A^613^ | | | 4 (1/25) | | 14.7 (5/34) | 0.0 (0/5) | 0.0 (0/30) | (6/94) |
| ***P. malariae* *dhfr* and *dhps* mutant genotype frequency; % (n)** | | | | | | | | | |
| Single dhfr | N^50^K^55^F^57^**R^58^**S^59^S^114^F^168^I^170^ | | | | 0.0 (0/26) | 0.0 (0/39) | 0.0 (0/9) | 21.4 (9/42) | 7.8 (9/116) |
| Double dhfr | N^50^K^55^**L^57^R^58^**S^59^S^114^F^168^I^170^ | | | | 84.6 (22/26) | 74.4 (29/39) | 100 (9/9) | 73.8 (31/42) | 78.4 (91/116) |
|  | N^50^K^55^F^57^**R^58^**S^59^**N^114^**F^168^I^170^ | | | | 0.0 (0/26) | 0.0 (0/39) | 0.0 (0/9) | 2.4 (1/42) | 0.9 (1/116) |
| Triple dhfr | N^50^K^55^**L^57^R^58^**S^59^**N^114^**F^168^I^170^ | | | | 0.0 (0/26) | 0.0 (0/39) | 0.0 (0/9) | 4.8 (2/42) | 1.7 (2/116) |
| Single dhps | S^436^**G^43^**^7^K^540^A^581^A^613^ | | | | 8 (2/25) | 11.8 (4/34) | 40 (2/5) | 6.7 (2/30) | 10.6 (10/94) |
| Double dhps | **S^436^G^43^**^7^K^540^A^581^A^613^ | | | | 56 (14/25) | 44.1 (15/34) | 0.0 (0/5) | 40 (12/30) | 37.2 (35/94) |
| **Combined** ***P. malariae dhfr* + *dhps* mutant genotype frequency; % (n)** | | | | | | | | | |
| Single | N^50^K^55^F^57^**R^58^**S^59^S^114^F^168^I^170^_ S^436^A^437^K^540^A^581^A^613^ | | | | 0.0 (0/25) | 0.0 (0/34) | 0.0 (0/5) | 10 (3/30) | 3.2 (3/94) |
|  | N^50^K^55^F^57^S^58^S^59^S^114^F^168^I^170^_ S^436^**G^437^**K^540^A^581^A^613^ | | | | 0.0 (0/25) | 2.9 (1/34) | 0.0 (0/5) | 0.0 (0/30) | 1.1 (1/94) |
| Double | N^50^K^55^**L^57^R^58^**S^59^S^114^F^168^I^170^_ S^436^A^437^K^540^A^581^A^613^ | | | | 40 (10/25) | 32.4 (11/34) | 60 (3/5) | 36.7 (11/30) | 37.2 (35/94) |
|  | N^50^K^55^F^57^**R^58^**S^59^**N^114^**F^168^I^170^_ S^436^A^437^K^540^A^581^A^613^ | | | | 0.0 (0/25) | 0.0 (0/34) | 0.0 (0/5) | 3.3 (1/30) | 1.1 (1/94) |
|  | N^50^K^55^F^57^S^58^S^59^S^114^F^168^I^170^_ **S^436^G^437^**K^540^A^581^A^613^ | | | | 8 (2/25) | 5.9 (2/34) | 0.0 (0/5) | 0.0 (0/30) | 4.3 (4/94) |
|  | N^50^K^55^F^57^**R^58^**S^59^S^114^F^168^I^170^_ S^436^**G^437^**K^540^A^581^A^613^ | | | | 0.0 (0/25) | 0.0 (0/34) | 0.0 (0/5) | 3.3 (1/30) | 1.1 (1/94) |
| Triple | N^50^K^55^F^57^**R^58^**S^59^S^114^F^168^I^170^_ **S^436^G^437^**K^540^A^581^A^613^ | | | | 0.0 (0/25) | 0.0 (0/34) | 0.0 (0/5) | 6.7 (2/30) | 2.1 (2/94) |
|  | N^50^K^55^**L^57^R^58^**S^59^S^114^F^168^I^170^_ S^436^**G^437^**K^540^A^581^A^613^ | | | | 4 (1/25) | 8.8 (3/34) | 40 (2/5) | 3.3 (1/30) | 7.4 (7/94) |
| Quadruple | N^50^K^55^**L^57^R^58^**S^59^S^114^F^168^I^170^_ **S^436^G^437^**K^540^A^581^A^613^ | | | | 32 (8/25) | 35.3 (12/34) | 0.0 (0/5) | 33.3 (10/30) | 31.9 (30/94) |
| Quintuple | N^50^K^55^**L^57^R^58^**S^59^**N^114^**F^168^I^170^_ **S^436^G^437^**K^540^A^581^A^613^ | | | | 0.0 (0/25) | 0.0 (0/34) | 0.0 (0/5) | 3.3 (1/30) | 1.1 (1/94) |

**Supplementary Table 6:** Genetic diversity of *Pfdhfr* isolates

|  | | | ***Pfdhfr*** | | | | | | |  |
| --- | --- | --- | --- | --- | --- | --- | --- | --- | --- | --- |
| **H/T stage** | | |  | **Genetic diversity indices** | | | | | |  |
| **Locality** | ***Anopheles*** | | **N** | **S** | **H** | **Hd** | **π** | **D** | **D*** |  |
| Bankeng | AGAM |  | 17 | 1 | 2 | 0.309 | 0.00052 | 0.08512 | 0.67700 |  |
| Elende | AFUN |  | 13 | 0 | 1 | 0.0 | 0.0 | 0.0 | 0.0 |  |
| Elon | AFUN |  | 5 | 0 | 1 | 0.0 | 0.0 | 0.0 | 0.0 |  |
| Gounougou | ACOL |  | 9 | 1 | 2 | 0.389 | 0.00065 | 0.15647 | 0.84040 |  |
| Mibellon | AFUN |  | 16 | 2 | 2 | 0.125 | 0.00042 | -1.49796 | -1.91470 |  |
| Simatou | ACOL |  | 10 | 0 | 1 | 0.0 | 0.0 | 0.0 | 0.0 |  |
| **Total** | **/** |  | **70** | **3** | **4** | **0.185** | **0.00044** | **-1.10111** | **-0.50428** |  |
| **Midgut stage** | |  | | | | | | | | |
| Bonaberi | ACOL |  | 2 | 0 | 1 | 0.0 | 0.0 | 0.0 | 0.0 |  |
| Elende | AFUN |  | 15 | 0 | 1 | 0.0 | 0.0 | 0.0 | 0.0 |  |
| Elon | AFUN |  | 15 | 3 | 2 | 0.343 | 0.0017 | 0.3416 | 1.0566 |  |
| Gounougou | ACOL |  | 13 | 0 | 1 | 0.0 | 0.0 | 0.0 | 0.0 |  |
| Mangoum | AGAM |  | 15 | 1 | 2 | 0.133 | 0.00022 | -1.1595 | -1.4265 |  |
| Mibellon | AFUN |  | 17 | 0 | 1 | 0.0 | 0.0 | 0.0 | 0.0 |  |
| Simatou | ACOL |  | 12 | 0 | 1 | 0.0 | 0.0 | 0.0 | 0.0 |  |
| Total | / |  | **89** | **3** | **3** | **0.088** | **0.00037** | **-1.1574** | **0.8368** |  |
| **Mixed stages** | |  | | | | | | | | |
| Obout | AFUN |  | 17 | 0 | 1 | 0.0 | 0.0 | 0.0 | 0.0 |  |
| **Blood stage (Humans)** | | | | | | | | | |  |
| Elende | **Human** |  | 20 | 1 | 2 | 0.189 | 0.00032 | -0.59155 | 0.64952 |  |
| Mibellon |  |  | 20 | 0 | 1 | 0.0 | 0.0 | 0.0 | 0.0 |  |
| Gounougou |  |  | 18 | 1 | 2 | 0.111 | 0.00019 | -1.16467 | -1.49949 |  |
| Total |  |  | **58** | **3** | **3** | **0.130** | **0.00033** | **-1.38578** | **-1.78934** |  |
| **Combined *Pfdhfr* seqs** | / |  | **234** | **3** | **5** | **0.115** | **0.00032** | **-0.96879** | **0.76146** |  |

**Supplementary Table 7:** Genetic diversity of the combined *Pfdhps* isolates

|  | | | ***Pfdhps*** | | | | | | |  |
| --- | --- | --- | --- | --- | --- | --- | --- | --- | --- | --- |
| **H/T stage** | | |  | **Genetic diversity indices** | | | | | |  |
| **Locality** | ***Anopheles*** | | **N** | **S** | **H** | **Hd** | **π** | **D** | **D*** |  |
| Bankeng | AGAM |  | 14 | 2 | 4 | 0.736 | 0.00133 | 1.74339 | 0.93543 |  |
| Elende | AFUN |  | 12 | 2 | 3 | 0.621 | 0.00121 | 1.22270 | 0.97295 |  |
| Elon | AFUN |  | 5 | 2 | 2 | 0.400 | 0.00103 | -0.97256 | -0.97256 |  |
| Gounougou | ACOL |  | 9 | 3 | 3 | 0.722 | 0.00186 | 1.17894 | 1.18844 |  |
| Mibellon | AFUN |  | 15 | 3 | 5 | 0.705 | 0.00130 | 0.28014 | 1.05657 |  |
| Simatou | ACOL |  | 10 | 3 | 3 | 0.689 | 0.00177 | 1.07659 | 1.15417 |  |
| **Total** | **/** |  | **65** | **3** | **6** | **0.680** | **0.00144** | **1.50189** | **0.86559** |  |
| **Midgut stage** | |  | | | | | | | | |
| Elende | AFUN |  | 15 | 3 | 4 | 0.619 | 0.00149 | 0.77143 | 1.05657 |  |
| Elon | AFUN |  | 14 | 3 | 3 | 0.275 | 0.00055 | -1.67053 | -2.09051 |  |
| Gounougou | ACOL |  | 12 | 1 | 2 | 0.167 | 0.00021 | -1.14053 | -1.32974 |  |
| Mangoum | AGAM |  | 14 | 2 | 3 | 0.692 | 0.00131 | 1.69598 | 0.93543 |  |
| Mibellon | AFUN |  | 17 | 2 | 2 | 0.309 | 0.00079 | 0.10997 | 0.89524 |  |
| Simatou | ACOL |  | 11 | 2 | 3 | 0.618 | 0.00136 | 1.66480 | 0.99697 |  |
| Total | / |  | **83** | **3** | **6** | **0.493** | **0.00109** | **0.75560** | **0.84301** |  |
| **Mixed stages** | |  | | | | | | | | |
| Obout | AFUN |  | 18 | 2 | 2 | 0.209 | 0.00054 | -0.68482 | 0.88460 |  |
| **Blood stage (Humans)** | | | | | | | | | |  |
| Elende | **Human** |  | 18 | 3 | 5 | 0.621 | 0.00123 | 0.28158 | -0.08478 |  |
| Mibellon |  |  | 20 | 3 | 6 | 0.574 | 0.00142 | 0.82012 | 1.00649 |  |
| Gounougou |  |  | 17 | 4 | 6 | 0.721 | 0.00198 | 0.93356 | 0.23149 |  |
| Total |  |  | **55** | **4** | **9** | **0.630** | **0.00152** | **0.79064** | **-0.12246** |  |
| **Combined *Pfdhps* seqs** | / |  | **221** | **4** | **9** | **0.576** | **0.00129** | **0.87397** | **-0.42748** |  |

**Supplementary Table 8:** Diversity index of pooled *Pmdhfr* sequence isolates

|  | | | ***Pmdhfr*** | | | | | | |  |
| --- | --- | --- | --- | --- | --- | --- | --- | --- | --- | --- |
| **H/T stage** | | |  | **Genetic diversity indices** | | | | | |  |
| **Locality** | ***Anopheles*** | | **N** | **S** | **H** | **Hd** | **π** | **D** | **D*** |  |
| Elende | AFUN |  | 6 | 2 | 2 | 0.333 | 0.00148 | -1.13197 | -1.15529 |  |
| Gounougou | ACOL |  | 5 | 2 | 2 | 0.600 | 0.00267 | 1.45884 | 1.45884 |  |
| Bankeng | AGAM |  | 4 | 0 | 1 | 0.0 | 0.0 | 0.0 | 0.0 |  |
| Mibellon | AFUN |  | 11 | 2 | 2 | 0.182 | 0.00081 | -1.42961 | -1.65766 |  |
| **Total** | **/** |  | **26** | **2** | **2** | **0.271** | **0.00120** | **0.07120** | **0.82564** |  |
| **Midgut stage** | |  | | | | | | | | |
| Elon | AFUN |  | 5 | 2 | 2 | 0.600 | 0.00267 | 1.45884 | 1.45884 |  |
| Elende | AFUN |  | 10 | 2 | 2 | 0.200 | 0.00089 | -1.40085 | -1.58662 |  |
| Gounougou | ACOL |  | 6 | 2 | 2 | 0.333 | 0.00148 | -1.13197 | -1.15529 |  |
| Mangoum | AGAM |  | 3 | 0 | 1 | 0.0 | 0.0 | 0.0 | 0.0 |  |
| Mibellon | AFUN |  | 12 | 2 | 2 | 0.409 | 0.00182 | 0.68788 | 0.97295 |  |
| Simatou | ACOL |  | 3 | 0 | 1 | 0.0 | 0.0 | 0.0 | 0.0 |  |
| **Total** | / |  | **39** | **2** | **2** | **0.391** | **0.00174** | **1.24449** | **0.77411** |  |
| **Mixed stages** | |  | | | | | | | | |
| Obout | AFUN |  | **9** | 0 | 1 | 0.0 | 0.0 | 0.0 | 0.0 |  |
| **Blood stage** | | | | | | | | | |  |
| Elende | **Human** |  | 15 | 4 | 5 | 0.476 | 0.00200 | -0.37646 | 1.15208 |  |
| Mibellon |  |  | 17 | 1 | 2 | 0.382 | 0.00071 | 0.56551 | 0.67700 |  |
| Gounougou |  |  | 10 | 2 | 3 | 0.511 | 0.00103 | -0.69098 | -0.28020 |  |
| Total |  |  | **42** | **4** | **5** | **0.434** | **0.00123** | **-0.66754** | **1.02191** |  |
| **Combined**  ***Pmdhfr* seq** | / |  | **116** | **4** | **6** | **0.369** | **0.00119** | **-0.27137** | **0.92807** |  |

|  | | | ***Pmdhps*** | | | | | | |  |
| --- | --- | --- | --- | --- | --- | --- | --- | --- | --- | --- |
| **H/T stage** | | |  | **Genetic diversity indices** | | | | | |  |
| **Locality** | ***Anopheles*** | | **N** | **S** | **H** | **Hd** | **π** | **D** | **D*** |  |
| Elende | AFUN |  | 6 | 2 | 2 | 0.6 | 0.00138 | 1.75324 | 1.27971 |  |
| Gounougou | ACOL |  | 5 | 4 | 4 | 0.9 | 0.00252 | 0.95707 | 0.95707 |  |
| Bankeng | AGAM |  | 4 | 5 | 4 | 1.0 | 0.00325 | 0.37186 | 0.37186 |  |
| Mibellon | AFUN |  | 10 | 2 | 4 | 0.733 | 0.00127 | 1.84427 | 1.02623 |  |
| **Total** | **/** |  | **25** | **6** | **9** | **0.757** | **0.00179** | **-0.05459** | **-0.99856** |  |
| **Midgut stage** | |  | | | | | | | | |
| Elon | AFUN |  | 5 | 2 | 2 | 0.6 | 0.00133 | 1.45884 | 1.45884 |  |
| Elende | AFUN |  | 10 | 3 | 4 | 0.8 | 0.00153 | 1.07659 | 1.15417 |  |
| Gounougou | ACOL |  | 5 | 2 | 2 | 0.6 | 0.00133 | 1.45884 | 1.45884 |  |
| Mangoum | AGAM |  | 3 | 1 | 2 | 0.7 | 0.00074 | 0.0 | 0.0 |  |
| Mibellon | AFUN |  | 11 | 2 | 3 | 0.564 | 0.00105 | 1.17621 | 0.99697 |  |
| **Total** | / |  | **34** | **3** | **4** | **0.656** | **0.00125** | **1.21732** | **0.93449** |  |
| **Mixed stages** | |  | | | | | | | | |
| Obout | AFUN |  | 7 | 1 | 2 | 0.600 | 0.00069 | 1.22474 | 1.22474 |  |
| **Blood stage** | | | | | | | | | |  |
| Elende | **Human** |  | 10 | 2 | 3 | 0.6 | 0.00115 | 1.33722 | 1.02623 |  |
| Mibellon |  |  | 10 | 2 | 3 | 0.6 | 0.00115 | 1.33722 | 1.02623 |  |
| Gounougou |  |  | 10 | 3 | 4 | 0.711 | 0.00138 | 0.47343 | 0.17464 |  |
| Total |  |  | **30** | **3** | **5** | **0.637** | **0.00125** | **1.03298** | **-0.26265** |  |
| **Combined**  ***Pmdhps* seq** | / |  | **94** | **7** | **11** | **0.675** | **0.00138** | **-0.28239** | **-0.53423** |  |

**Supplementary Table 9:** Genetic diversity statistics of the pooled *Pmdhps* isolates

**Legend:** N = number of sequences, H = number of distinct haplotype, S = polymorphic sites, Hd = haplotype diversity, π = nucleotide diversity (the average number of nucleotide differences per site between two sequences)
